# HEXACO personality dimensions as predictors of environmental attitudes, socio-moral orientations, and climate change beliefs

**DOI:** 10.1016/j.isci.2025.113753

**Published:** 2025-10-17

**Authors:** Marianna Drosinou, Jussi Palomäki, Anton Kunnari, Mika Koverola, Markus Jokela, Michael Laakasuo

**Affiliations:** 1 University of Helsinki, Faculty of Medicine, Discipline of Psychology, Helsinki, Finland; 2 University of Turku, Faculty of Social Sciences, Department of Psychology, Turku, Finland; 3 Finnish Institute for Health and Welfare, Department of Public Health and Welfare, Helsinki, Finland; 4 University of Helsinki, Faculty of Arts, Cognitive Science Unit, Helsinki, Finland; 5 University of Turku, Faculty of Social Sciences, Department of Social Research, Turku, Finland

**Keywords:** environmental science, environmental policy, environmental issues, social sciences, psychology

## Abstract

In this article, we investigate which personality dimensions provide a common psychological basis for environmental measures and socio-moral orientations (e.g., feeling connected to others, perceiving the environmental problem as moral). In a cross-sectional study (*N* = 634), we explored the associations between the HEXACO dimensions and a wide range of environmental measures as well as socio-moral orientations related to environmentalism. We found that Openness to Experience was the most prominent predictor of environmental measures, socio-moral orientations, and the belief that climate change is caused by human activity. We further provide a comprehensive overview of how each personality dimension connects socio-moral orientations and environmental measures, highlighting the multifaceted nature of environmentalism. The findings underscore the role of individual differences in coping with the environmental crisis. Our article replicates and extends previous research, contributing to the ongoing discussion on how differences in individual predispositions influence environmental engagement.

## Highlights

• Openness to Experience is associated with a large number of environmental measures
• People high in Openness are also high in environment-related socio-moral orientations
• Climate change beliefs are also associated with high Openness to Experience

## Graphical abstract

**Figure:**
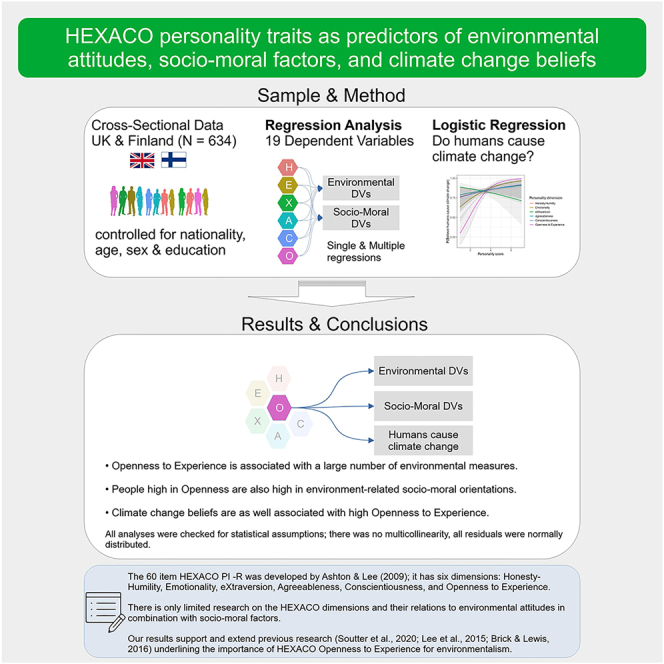

## Introduction

Accumulating evidence highlights the ecological challenge we face as a species. We have now reached a critical point where environmental overexploitation is globally recognized, and significant effort is needed to restore the already incurred damage. Climate change has many alarming global environmental effects: temperature and sea levels are rising, oceans are warming, and ice masses are decreasing; extreme weather conditions are more frequent than before, and there have been significant reductions in biodiversity.^1^ The Intergovernmental Panel on Climate Change^2^ concluded that there is a higher than 95% probability that human activity has been the cause of our planet warming over the last 50 years. Nevertheless, many still deny that climate change is happening, and among those who recognize it, many think it has no link to human activity. A substantial amount of research has explored what causes people to behave harmfully toward the environment, although we still do not fully understand the reasons. Therefore, a better understanding of the psychological factors related to environmental attitudes could improve the situation.

### Connectedness and socio-moral orientations

Human disconnection from nature has been proposed to be one of the main causes of the ecological crisis which involves a moral dimension^3^ in addition to cognitive and emotional dimensions. Early environmentalists argued that all living organisms are interdependent and form a community consisting of people, other animals, and plants. Aldo Leopold^4^ suggested that a sense of connectedness to nature is necessary in addressing the environmental challenge. Leopold proposed a *land ethic* where the boundaries of the community in which humans belong extend to collectively include the land (i.e., animals, plants, waters, and so forth). Recent research by Rottman and colleagues^5^ showed that the moral valuations of people and nature are positively related; people typically expand their perceived inner moral circle (i.e., the targets of moral value attribution) to first include other people (outgroups) and then nature (animals and the natural world), while some people prioritize nature in their moral circle before other people. In other words, a sense of ecological connectedness, namely the realization of interconnectedness of all life, is a crucial factor in understanding the way we think and act toward the environment, and in raising moral awareness regarding this issue. Recent findings showed that feelings of connectedness to nature and people are associated with environmental attitudes and ecologically conscious consumer behavior, and that the associations are mediated through moral awareness of the environmental issue.^6^

According to Schultz,^7^ the consideration of similarities between people and nature is the core of environmental ethics, and the human responsibility of protecting the environment stems from their perceived symbiotic connection with nature. However, if people perceive they are separate from nature, there is no apparent moral responsibility. Previous findings suggest that a sense of interdependence with nature can enhance eco-friendly behaviors; in fact, feeling connected to nature may be one of the most important elements related to environmentalism.^8^^,^^9^^,^^10^^,^^11^ Moreover, Mayer and Frantz^12^ found that connectedness to nature predicts ecological behavior and increased subjective well-being. In addition, extending one’s own identity to include the natural world increased the likelihood of environmentally friendly behavior,^13^ and taking the perspective of an animal induced greater concern for the animal.^14^

Similarly, including other people in one’s own sense of self appears to increase relationship closeness,^15^ which, in turn, enhances empathy, perspective-taking, cooperation and willingness to help others.^16^ Consequently, feeling connected to other people can increase prosocial behavior, such as environmental behavior, which is positively linked with moral norms and moral obligation.^17^ Previous work has also suggested that feeling related to humanity globally is relevant for pro-environmental behaviors,^18^ and people who felt more as part of the human community globally (rather than nationally) were more likely to believe that humans contribute to climate change.^19^ Finally, previous evidence suggests that taking the perspective of people in the future increases sustainable decision-making, highlighting the influence of neural basis in sustainable behavior.^20^

### Value-Belief-Norm (VBN) model

Overall, there are three types of environmental concerns; egoistic, altruistic, and biospheric,^10^ and pro-environmental behavior is a mixture of self-interest and concern for other people, other species, or whole ecosystems.^21^ These proposed subtypes are in line with the Value-Belief-Norm model (VBN),^22^^,^^23^ which proposes that there are three orientations or ethics for environmentalism: egoistic, altruistic (social), and biospheric. Based on the VBN model, personal environmental norms develop in reaction to potential negative impacts on valued objects. When individuals have an egoistic orientation, their environmental concern stems from perceived risks to themselves. In contrast, a social-altruistic orientation motivates concern based on potential harm to other people, while a biospheric orientation reflects concern for the well-being of all living organisms. Although these value orientations are conceptually distinct, they tend to be interrelated.^24^

These three value orientations have also been found to be important for climate change beliefs. The results of a meta-analysis on climate change beliefs showed that concern about the environment’s vulnerability was strongly associated with belief in climate change.^25^ In addition, results confirmed that biospheric values (i.e., placing a high importance on the natural environment) was associated with the belief that climate change is real.^25^ This finding is in accordance with the Climate Change Risk Perception Model (CCRPM)^26^ which identifies the importance of cognitive, experiential and socio-cultural factors in climate change risk pereception. In this model, the three value orientations (i.e., egoistic, altruistic, and biospheric) contribute to a better understanding of risk perception.

### Past research

Most environmental research on individual differences has rarely focused on such *socio-moral orientations* (e.g., feeling connected to other people, or perceiving the environmental problem as a moral one) when investigating environmental attitudes and behaviors in conjunction with personality. Previous work on personality and environmental measures has been mainly focused on the Big Five framework, while recent developments involving the HEXACO model^27^ have received much less attention. Furthermore, personality and psychometric measures on environmentally oriented attitudes and behaviors are rarely investigated together extensively. There is also a lack of evidence on the associations between personality traits and climate change beliefs.^28^^,^^29^ In this article, we explore in detail the associations between HEXACO personality dimensions, socio-moral orientations pertaining to the environment, and an extensive number of environmental measures along with climate change beliefs. Thus, we aim to fill a gap in the previous literature by focusing on HEXACO rather than Big Five, and by further examining socio-moral orientations together with environmental measures.

Previous environmental research on personality reports that Big Five Agreeableness and Openness to Experience predict pro-environmental values,^30^ and greater environmental concern^31^ – however, it is not clear whether there is a difference in general prosocial orientation and distinct environmental values. Only a handful of articles have looked at the effects of HEXACO personality dimensions on environmental measures.^32^^,^^33^^,^^34^^,^^35^^,^^36^^,^^37^ For instance, Markowitz et al.^33^ examined personality dimensions from four different personality inventories in relation to pro-environmental actions; among the dimensions, Openness to Experience was found to have a consistent positive association with pro-environmental activities (e.g., recycling, using public transportation, buying organic food, and so forth), and that effect was mediated by environmental attitudes and connection to nature. Moreover, Hilbig and colleagues^35^ demonstrated that Honesty-Humility is linked to environmental attitudes and behavior, partly due to individual differences in prosocial orientation, thus bridging personality dimensions and ecological behavior. Furthermore, Lee et al.^36^ found a correlation between connectedness to humanity and connectedness to nature, and further showed that Honesty-Humility and Openness to Experience were the primary personality correlates of both of these constructs. They also showed that the effect of the latter personality dimensions on pro-environmental attitudes and behaviors was mediated by connectedness to nature and connectedness to humanity. Finally, Panno and colleagues^38^ found that Openness to Experience and Honesty-Humility were the most prominent dimensions in predicting pro-environmental behavior via moral anger; while Openness to Experience was also the most prominent dimension in predicting climate change action via moral anger.

### HEXACO links to socio-moral orientations and environmental measures

Based on previous findings, we assume certain links between personality dimensions and socio-moral orientations and environmental measures, expecting that Openness will be the most prominent predictor in our analyses.^33^^,^^36^^,^^38^ Individuals high in Openness are likely to identify with all humanity and the natural world,^36^ and attend to it.^39^ In addition, they show appreciation for nature, and are characterized by high creativity,^40^^,^^41^ which has been further associated with future-oriented thinking.^42^ Therefore, it is possible that these individuals can more vividly imagine the consequences of climate change and motivate their care toward environmental issues. Moreover, individuals high in Openness are naturally inclined toward knowledge^32^ and may thus have acquired an evidence-based understanding of the consequences of environmental degradation. Finally, they are often the first to adopt new habits,^40^ which makes them more inclined to engage in environmental activities, as there is a general societal tendency to turn to more green practices. Thus, mechanisms such as appreciation for nature and future orientation can underline the relation of Openness to environmentalism.

Individuals with high Emotionality are empathetic and caring^40^ and, similar to individuals high in Openness, they are likely to identify with all humanity.^36^ Thus, they would be expected to adopt environmental attitudes due to their sensitivity, and to respond to environmental threats related to the survival of other species, other people, and themselves. Moreover, individuals with high Emotionality are characterized by high alertness to physical and social threat-related cues and experience anxiety in response to life stresses associated with survival.^40^^,^^41^ It is possible that empathy can be the underlying mechanism for the link of Emotionality to environmentalism.

Extraverts, in turn, would be expected to engage in environmental and social actions that involve a (social) reward component, particularly those involving social interactions and recognition.^32^ As extraverted individuals have a natural inclination toward sociability and external stimulation, they may pursue social approval, or other forms of social rewards.^43^^,^^44^

Individuals high in Honesty-Humility have altruistic values and feel concern for other people.^36^ Therefore, they would be expected to engage in environmental attitudes^32^^,^^37^ due to their prosocial values^35^ and moral orientation.^36^ Therefore, value internalization could possibly explain the relation of Honesty-Humility to environmentalism.

Individuals with high Agreeableness are also characterized by prosocial motivations.^45^ Thus, they may adopt environmental attitudes as a way to please, cooperate with, and help others.

Finally, individuals with high Conscientiousness follow social norms and have a strong sense of duty, which may motivate certain environmental behaviors.^34^ However, some findings do not support a strong link between Conscientiousness and environmental measures,^33^^,^^35^ indicating that there is no intrinsic motivational structure associated with Conscientiousness, which would drive people with high Conscientiousness toward environmentalism. However, should there be strong social norms or laws toward individual obligation to the conservation of nature, this could then (socially) motivate high Conscientiousness individuals toward pro-environmental behaviors.

### Current study

In this study, we identify the personality dimensions underlying the positive and contrasting relationships between socio-moral orientations and environmental measures. By answering the question of how specific personality dimensions bridge or separate socio-moral orientations and environmental attitudes and behaviors, this study highlights the need to consider the multifaceted nature of environmentalism and provides a better understanding of the psychological mechanisms that promote it. Moreover, insights can advance current knowledge on the relationship between connectedness, moral awareness, and environmental engagement, elaborating on previous research.^7^

We include measures of social awareness, connectedness, and moral orientations, and we investigate these orientations in conjunction with several environmental attitudinal and behavioral measures (self-reported), as these allow contrasting socially psychologically oriented findings with more environmental psychological perspectives. Based on previous findings, we additionally propose that a sense of connectedness to one’s own self (feelings, motives, and so forth) is a necessary requirement for individuals to expand this notion to other people and to nature, and thus a potential factor in adopting ecological attitudes and behaviors. Feelings of interconnectedness to one’s surroundings naturally require people to also be aware of themselves, since connectivity to nature is defined as “a perception of sameness between the self, others, and the natural world.”^9^ Therefore, we also include in our study constructs such as self-awareness and mindfulness.

In this article, we aim to extend previous literature to get a detailed and comprehensive picture of how personality is linked to environmentalism. First, we examine the associations between HEXACO and multiple environmental measures. Second, we examine the associations between HEXACO and different socio-moral orientations that are known to promote pro-environmental attitudes and behaviors. Finally, we examine how the HEXACO personality dimensions relate to climate change beliefs. We expect Openness to Experience to be the most prominent dimension in predicting environmental attitudes and self-reported buying behavior. We also assume that Openness will be associated with the socio-moral orientations and climate change beliefs. We further explore how the other personality dimensions associate with our measures.

### Study

We examine the associations of personality, socio-moral orientations related to the environment, environmental attitudes and behavior, as well as climate change beliefs. The main objective is to find which HEXACO personality dimensions contribute the most to eco-friendly attitudes and self-reported behavior, connectedness (nature, people, self), and other moral orientations (i.e., acknowledging a moral aspect in environmental issues). We focus on the personality dimensions that connect socio-moral and environmental tendencies. We also include measures of feeling connected to one’s self, moral awareness, and moral obligation related to protecting the environment. We seek to replicate previous findings and add to the existing literature by employing different measures of socio-moral orientations together with environmental measures.

## Results

### Simple and multiple regressions

We first analyzed the direct uncontrolled associations with simple regressions (see Table 1) and then ran regressions where all HEXACO dimensions were entered into the model simultaneously (see Table 2).

**Table 1:** Simple direct regression associations between HEXACO dimensions and dependent variables (*N* = 634)

Predictors	Mindful Attention Awareness	Social Awareness of Self	Social Awareness of Others	Inclusion of Other in Self	Inclusion of Nature in Self	Moral Awareness	Moral Obligation	Environmental Self-identity	Environmental Viewpoints	General Concern	Biospheric Concern	Social Concern	Egoistic Concern	New Ecological Paradigm	Awareness of Consequences -General	Awareness of Consequences - Biospheric	Awareness of Consequences - Social	Awareness of Consequences - Egoistic	Eco-conscious Consumer Behavior
	B [95% CI]	B [95% CI]	B [95% CI]	B [95% CI]	B [95% CI]	B [95% CI]	B [95% CI]	B [95% CI]	B [95% CI]	B [95% CI]	B [95% CI]	B [95% CI]	B [95% CI]	B [95% CI]	B [95% CI]	B [95% CI]	B [95% CI]	B [95% CI]	B [95% CI]
Honesty-Humility	**0.34 [0.21, 0.47]**^c^	−0.07 [−0.17, 0.02]	0.12 [0.02, 0.23]^a^	0.05 [−0.13, 0.24]	0.23 [0.02, 0.44]^a^	**0.26 [0.11, 0.41]**^c^	**0.28 [0.15, 0.40]**^c^	**0.22 [0.11, 0.33]**^c^	**0.29 [0.15, 0.43]**^c^	0.05 [−0.07, 0.18]	**0.33 [0.17, 0.49]**^c^	**0.31 [0.15, 0.46]**^c^	**−0.47 [−0.64, -0.30]**^c^	**0.34 [0.21, 0.47]**^c^	**0.31 [0.20, 0.42]**^c^	**0.30 [0.18, 0.42]**^c^	**0.29 [0.16, 0.42]**^c^	**0.35 [0.23, 0.46]**^c^	**0.32 [0.15, 0.48]**^c^
Emotionality	**−0.22 [−0.36, −0.09]**^c^	**0.22 [0.11, 0.32]**^c^	**0.42 [0.31, 0.53]**^c^	0.29 [0.08, 0.49]^b^	−0.01 [−0.23, 0.20]	**0.26 [0.10, 0.42]**^c^	0.06 [−0.06, 0.20]	0.05 [−0.06, 0.17]	0.19 [0.03, 0.34]^a^	**0.34 [0.21, 0.47]**^c^	**0.43 [0.26, 0.59]**^c^	**0.39 [0.23, 0.56]**^c^	0.21 [0.02, 0.39]^a^	0.19 [0.05, 0.33]^b^	0.13 [0.01, 0.25]^a^	0.07 [−0.05, 0.21]	0.17 [0.03, 0.31]^a^	0.15 [0.02, 0.27]^a^	0.03 [−0.14, 0.20]
Extraversion	**0.25 [0.13, 0.37]**^c^	0.08 [−0.00, 0.18]	**0.20 [0.10, 0.30]**^c^	**0.95 [0.79, 1.11]**^c^	**0.37 [0.18, 0.56]**^c^	0.09 [−0.04, 0.23]	0.17 [0.06, 0.29]^b^	**0.22 [0.12, 0.33]**^c^	−0.02 [−0.16, 0.10]	**0.25 [0.13, 0.37]**^c^	0.08 [−0.06, 0.23]	**0.42 [0.28, 0.56]**^c^	0.25 [0.09, 0.41]^b^	−0.03 [−0.15, 0.08]	0.03 [−0.07, 0.13]	−0.05 [−0.16, 0.06]	0.05 [−0.06, 0.17]	0.09 [−0.01, 0.20]	0.24 [0.09, 0.39]^b^
Agreeableness	**0.24 [0.11, 0.38]**^c^	0.06 [−0.04, 0.16]	**0.32 [0.21, 0.43]**^c^	0.32 [0.12, 0.52]^b^	0.23 [0.01, 0.45]^a^	0.14 [−0.01, 0.30]	0.19 [0.06, 0.33]^b^	**0.26 [0.14, 0.38]**^c^	0.15 [−0.00, 0.30]	0.14 [0.00, 0.28]^a^	0.21 [0.04, 0.38]^a^	**0.36 [0.19, 0.53]**^c^	−0.14 [−0.32, 0.04]	0.13 [−0.01, 0.27]	0.12 [0.01, 0.24]^a^	0.10 [−0.03, 0.23]	0.15 [0.01, 0.29]^a^	0.13 [0.00, 0.25]^a^	**0.36 [0.19, 0.54]**^c^
Conscientiousness	**0.62 [0.49, 0.75]**^c^	**0.17 [0.07, 0.28]**^c^	−0.02 [−0.13, 0.08]	0.17 [−0.02, 0.37]	0.23 [0.01, 0.44]^a^	0.07 [−0.07, 0.23]	0.14 [0.01, 0.28]^a^	0.12 [0.00, 0.24]^a^	0.20 [0.05, 0.35]^b^	0.02 [−0.10, 0.16]	0.09 [−0.07, 0.26]	0.08 [−0.08, 0.25]	−0.09 [−0.27, 0.09]	0.11 [−0.02, 0.25]	**0.24 [0.12, 0.35]**^c^	0.20 [0.07, 0.33]^b^	**0.24 [0.10, 0.37]**^c^	**0.28 [0.16, 0.40]**^c^	0.15 [−0.01, 0.33]
Openness	**0.32 [0.19, 0.44]**^c^	**0.36 [0.27, 0.46]**^c^	**0.25 [0.15, 0.35]**^c^	0.24 [0.05, 0.43]^b^	**0.56 [0.36, 0.76]**^c^	**0.47 [0.33, 0.62]**^c^	**0.50 [0.38, 0.62]**^c^	**0.38 [0.27, 0.49]**^c^	**0.55 [0.41, 0.69]**^c^	**0.25 [0.13, 0.38]**^c^	**0.53 [0.37, 0.68]**^c^	**0.36 [0.21, 0.52]**^c^	−0.12 [−0.29, 0.05]	**0.42 [0.29, 0.54]**^c^	**0.52 [0.42, 0.62]**^c^	**0.54 [0.42, 0.66]**^c^	**0.49 [0.37, 0.61]**^c^	**0.53 [0.42, 0.64]**^c^	**0.53 [0.37, 0.69]**^c^

a *p* < .05.

b *p* < .01.

c *p <=* .001; Bolded font: *p* ≤.001; the strongest absolute association (B value) per variable is underlined; the analyses have been controlled for demographic variables. R^2^ values range from .01 to .21; for the full list of R^2^ values, see Table S1.

**Table 2:** Multiple regression associations between HEXACO dimensions and dependent variables (*N* = 634)

	Mindful Attention Awareness	Social Awareness of Self	Social Awareness of Others	Inclusion of Other in Self	Inclusion of Nature in Self	Moral Awareness	Moral Obligation	Environmental Self-identity	Environmental Viewpoints	General Concern	Biospheric Concern	Social Concern	Egoistic Concern	New Ecological Paradigm	Awareness of Consequences -General	Awareness of Consequences - Biospheric	Awareness of Consequences - Social	Awareness of Consequences - Egoistic	Eco-conscious Consumer Behavior
	B [95% CI]	B [95% CI]	B [95% CI]	B [95% CI]	B [95% CI]	B [95% CI]	B [95% CI]	B [95% CI]	B [95% CI]	B [95% CI]	B [95% CI]	B [95% CI]	B [95% CI]	B [95% CI]	B [95% CI]	B [95% CI]	B [95% CI]	B [95% CI]	B [95% CI]
R^2^	0.20	0.19	0.24	0.24	0.09	0.18	0.19	0.15	0.19	0.13	0.17	0.20	0.09	0.17	0.24	0.23	0.15	0.26	0.14

**Predictors**																			

Honesty-Humility	**0.25 [0.12, 0.38]**^c^	**−0.20 [−0.30, −0.09]**^c^	−0.00 [−0.10, 0.09]	0.03 [−0.15, 0.21]	0.13 [−0.08, 0.35]	0.15 [−0.00, 0.31]	0.18 [0.05, 0.31]^b^	0.12 [0.00, 0.24]^a^	0.14 [0.00, 0.29]^a^	−0.20 [−0.15, 0.11]	0.19 [0.02, 0.35]^a^	0.20 [0.04, 0.36]^a^	**−0.45 [−0.63, −0.27]**^c^	**0.25 [0.11, 0.38]**^c^	**0.20 [0.09, 0.31]**^c^	0.18 [0.06, 0.31]^b^	0.17 [0.03, 0.30]^a^	**0.24 [0.12, 0.35]**^c^	0.18 [0.01, 0.35]^a^
Emotionality	−0.19 [−0.31, −0.06]^b^	**0.28 [0.17, 0.38]**^c^	**0.51 [0.40, 0.61]**^c^	**0.55 [0.37, 0.73]**^c^	0.07 [−0.14, 0.29]	**0.29 [0.14, 0.45]**^c^	0.11 [−0.02, 0.24]	0.11 [−0.00, 0.23]	0.19 [0.04, 0.33]^b^	**0.43 [0.30, 0.56]**^c^	**0.46 [0.30, 0.63]**^c^	**0.52 [0.36, 0.67]**^c^	**0.32 [0.14, 0.50]**^c^	0.18 [0.04, 0.31]^b^	0.14 [0.03, 0.25]^b^	0.06 [−0.06, 0.19]	0.19 [0.06, 0.32]^b^	0.17 [0.05, 0.28]^b^	0.09 [−0.07, 0.26]
Extraversion	0.12 [0.00, 0.24]^a^	0.04 [−0.04, 0.14]	**0.25 [0.16, 0.34]**^c^	**1.03 [0.87, 1.20]**^c^	0.31 [0.11, 0.51]^b^	0.09 [−0.04, 0.24]	0.14 [0.02, 0.26]^a^	**0.19 [0.08, 0.30]**^c^	−0.07 [−0.20, 0.05]	**0.30 [0.18, 0.42]**^c^	0.11 [−0.03, 0.26]	**0.49 [0.34, 0.63]**^c^	**0.30 [0.14, 0.47]**^c^	−0.03 [−0.16, 0.08]	−0.01 [−0.11, 0.09]	−0.11 [−0.22, 0.00]	0.01 [−0.10, 0.13]	0.06 [−0.04, 0.16]	0.18 [0.03, 0.34]^a^
Agreeableness	0.06 [−0.06, 0.20]	0.08 [−0.02, 0.18]	**0.30 [0.19, 0.41]**^c^	0.18 [−0.01, 0.37]	0.09 [−0.13, 0.32]	0.05 [−0.10, 0.22]	0.07 [−0.05, 0.21]	0.17 [0.04, 0.29]^b^	0.06 [−0.08, 0.22]	0.11 [−0.02, 0.25]	0.11 [−0.05, 0.28]	0.23 [0.07, 0.40]^b^	−0.01 [−0.20, 0.17]	0.02 [−0.11, 0.16]	0.01 [−0.09, 0.13]	0.00 [−0.13, 0.13]	0.05 [−0.08, 0.19]	−0.00 [0.12, 0.11]	0.24 [0.06, 0.42]^b^
Conscientiousness	**0.52 [0.39, 0.64]**^c^	0.13 [0.03, 0.23]^a^	−0.13 [−0.23, −0.02]^a^	−0.05 [−0.24, 0.12]	0.05 [−0.16, 0.27]	−0.03 [−0.19, 0.12]	0.00 [−0.12, 0.14]	−0.00 [−0.12, 0.11]	0.10 [−0.04, 0.25]	−0.06 [−0.19, 0.06]	−0.03 [−0.20, 0.13]	−0.10 [−0.25, 0.05]	−0.06 [−0.24, 0.12]	0.02 [−0.11, 0.15]	0.13 [0.02, 0.24]^a^	0.10 [−0.01, 0.23]	0.13 [0.00, 0.26]^a^	0.16 [0.04, 0.27]^b^	−0.01 [−0.18, 0.16]
Openness	0.16 [0.04, 0.28]^b^	**0.37 [0.28, 0.47]**^c^	**0.23 [0.14, 0.33]**^c^	0.10 [−0.06, 0.27]	**0.48 [0.27, 0.69]**^c^	**0.45 [0.30, 0.59]**^c^	**0.45 [0.33, 0.57]**^c^	**0.32 [0.21, 0.43]**^c^	**0.52 [0.38, 0.66]**^c^	**0.23 [0.11, 0.36]**^c^	**0.49 [0.34, 0.65]**^c^	**0.27 [0.12, 0.42]**^c^	−0.06 [−0.23, 0.10]	**0.38 [0.26, 0.51]**^c^	**0.47 [0.37, 0.58]**^c^	**0.51 [0.39, 0.63]**^c^	**0.44 [0.32, 0.57]**^c^	**0.46 [0.35, 0.57]**^c^	**0.46 [0.29, 0.62]**^c^
Nationality	−0.06 [−0.23, 0.10]	−0.14 [−0.27, −0.06]^a^	0.15 [0.01, 0.28]^a^	0.48 [0.24, 0.72]^c^	0.46 [0.17, 0.75]^b^	−0.44 [−0.65, −0.24]^c^	−0.28 [−0.46, −0.11]^c^	−0.09 [−0.25, 0.06]	−0.35 [−0.55, −0.16]^c^	0.05 [−0.11, 0.23]	−0.02 [−0.24, 0.19]	−0.06 [−0.26, 0.14]	0.25 [0.01, 0.49]^a^	−0.16 [−0.34, 0.01]	−0.22 [−0.37, −0.08]^b^	−0.40 [−0.57, −0.23]^c^	−0.02 [−0.20, 0.14]	−0.24 [−0.39, −0.09]^c^	0.00 [−0.22, 0.22]

a *p* < .05.

b *p* < .01.

c *p <=* .001; Bolded font: *p* ≤.001; the strongest absolute association (B value) per variable is underlined; the analyses have been controlled for demographic variables. For the semi-partials, see Table S2.

In the direct regression analyses, Openness was reliably associated with 17 of the DVs (all Bs > 0.25, *p* ≤ 0.001), and had the strongest associations (compared with the other HEXACO dimensions) with 13 DVs, including both socio-moral orientations (e.g., Self-Awareness, Moral Awareness, Moral Obligation) and environmental measures (e.g., Environmental Viewpoints, NEP, ECCB). These results remain unchanged when controlling for the effects of the other HEXACO dimensions (except for MAAS, where the association was at the *p* < 0.01 -level in the controlled associations). Thus, the effect of Openness to Experience is the strongest predictor of socio-moral orientations as well as environmental attitudes (Tables 1 and 2). This finding is in line with previous literature.^32^^,^^33^^,^^34^ People with high Openness are more likely to have an environmental self-identity, feel interconnected with nature, be morally aware of the environmental issue, feel a moral obligation to protect the environment, and also have environmental viewpoints, be concerned about nature, be aware of the consequences of environmental degradation, and engage in eco-conscious buying.

Emotionality was found to have a reliable association with 7 DVs (all |B|s > 0.22, *p* ≤ 0.001) in the direct regressions, and after controlling for the other dimensions, reliable associations for Emotionality were found with 8 DVs (all |B|s > 0.28, *p ≤* 0.001); it was associated with socio-moral orientations (Self- and Other- Awareness, IOS, Moral Awareness), and Concern (General, Biospheric, Social, Egoistic). People high in Emotionality are more likely to be aware of other people’s needs, feel interconnected to them, be morally aware of environmental issues, as well as more likely to be concerned about environmental problems on all levels.

Honesty-Humility was reliably associated with 14 of the DVs (all |B|s > 0.22, *p ≤* 0.001; see Table 1) in the direct regressions. However, after controlling for the other dimensions, there were 6 reliable associations (all |B|s > 0.20, *p ≤* 0.001), including two negative associations with Self-awareness and Egoistic Concern (Table 2). The reliable negative association of Honesty-Humility with Egoistic Concern is consistent in both simple and multiple regression analyses. In addition, we found a reliable association with awareness of negative consequences regarding one’s self in both analyses. In other words, people high in Honesty-Humility are not concerned about themselves, although they are highly aware of the negative consequences that environmental degradation could have for them. Honesty-Humility also predicted NEP; since NEP reflects that the environment is valuable on its own and it does not exist to serve human needs, it is sensible that humbler people are more accepting of this view. Thus, Honesty-Humility was implicated in socio-moral and environmental measures, but the effects were less prominent compared to Openness.

Extraversion was reliably associated with 7 DVs (all Bs > 0.20, *p ≤* 0.001) in the direct regressions. After controlling for the other dimensions, Extraversion was associated with 6 DVs (all Bs > 0.19, *p ≤* 0.001): Environmental Self-identity, Other-Awareness, IOS, Concern (General, Social, Egoistic). Extraverts’ general environmental concern was driven by their focus on the social aspect. Extraversion (similar to Emotionality) was reliably associated with socio-moral ecological orientations (i.e., being aware of others, feeling interconnected to others), as well as feeling concern about people in both direct and controlled analyses. It seems that extraverted individuals focus their environmental concern on people.

Agreeableness was associated with 5 DVs (all Bs > 0.24, *p ≤* 0.001) in the direct regressions, but after controlling for the other dimensions, only one reliable association (B = 0.30, *p ≤* 0.001) with Other-awareness remained, which is not surprising given the nature of agreeable people.

In the direct regressions, Conscientiousness had 5 reliable associations (all Bs > 0.17, *p ≤* 0.001) with the DVs, but after controlling for the other personality dimensions, it was reliably associated only with MAAS (B = 0.52, *p ≤* 0.001).

Overall, Openness to Experience was the most important dimension in predicting both socio-moral and environmental tendencies to a large extent (As a robustness check, we also re-analyzed the data with *lavaan* R package^46^; we regressed all the HEXACO dimensions on all the DVs simultaneously, which amounts to a fully saturated model (where all the DVs were allowed to correlate). In this model (and its further refinements), all the B-values remained the same as those reported in Table 2).

### Climate change beliefs

The climate change beliefs questions were analyzed separately from the other DVs, since some of the nominal response categories (see Table S3 for item descriptions) in the individual datasets had uneven and low numbers of responses (see later in discussion). We analyzed the effects of all the HEXACO dimensions on each of these DVs with multiple regressions controlling for the effects of nationality, age, sex, and education (Table 3). The nominal climate change questions were analyzed with multinomial logistic regressions (categorical DVs: questions 1 and 3), while the continuous climate change questions (continuous slider DVs: questions 2 and 4) were analyzed with OLS regressions.

**Table 3:** Climate change-related questions and HEXACO – Controlled Associations (*N* = 634)

	Independent Variables/Predictors					
	Honesty-Humility	Emotionality	Extraversion	Agreeableness	Conscientiousness	Openness
	OR/B [95% CI]	OR/B [95% CI]	OR/B [95% CI]	OR/B [95% CI]	OR/B [95% CI]	OR/B [95% CI]
**Dependent Variables**						

1. Which of the following best represents your thoughts about climate change?						
a) I do not think it is happening	1.00 (ref)	1.00 (ref)	1.00 (ref)	1.00 (ref)	1.00 (ref)	1.00 (ref)
b) I do not know if it is happening	1.19 [0.28, 5.12]	2.93 [0.74,11.65]	1.42 [0.42, 4.77]	1.32 [0.28, 6.09]	0.86 [0.24, 3.07]	1.62 [0.42, 6.24]
c) It is happening, but it is a natural variation	1.01 [0.24, 4.22]	1.79 [0.46, 6.93]	1.57 [0.47, 5.17]	1.36 [0.30, 6.19]	1.41 [0.40, 4.97]	2.20 [0.58, 8.32]
d) It is happening, and humans are the cause	1.39 [0.35, 5.49]	3.42 [0.94, 12.48]	1.19 [0.38, 3.68]	1.72 [0.40, 7.32]	1.42 [0.43, 4.63]	4.50 [1.26, 15.96]^a^
a + b + c vs. d	1.27 [0.87, 1.85]	1.62 [1.11, 2.37]^a^	0.82 [0.58, 1.15]	1.30 [0.87, 1.94]	1.24 [0.84, 1.83]	**2.45 [1.69, 3.56]**^b^
2. How certain are you that humans contribute to climate change?	1.03 [−1.76, 3.83]	2.98 [0.18, 5.78]^a^	1.06 [−1.47, 3.60]	−0.67 [−3.61, 2.25]	−0.37 [−3.19, 2.44]	**5.95 [3.29, 8.60]**^b^
3. Who should have the main responsibility?						
a) International Organizations	1.17 [0.67, 2.05]	1.27 [0.72, 2.26]	0.84 [0.51, 1.38]	0.94 [0.52, 1.71]	1.24 [0.70, 2.18]	1.29 [0.77, 2.17]
b) National Government	1.21 [0.75, 1.95]	0.84 [0.52, 1.37]	1.00 [0.65, 1.53]	1.13 [0.68, 1.88]	0.82 [0.50, 1.33]	0.97 [0.62, 1.51]
c) Local Government	1.12 [0.39, 3.24]	0.61 [0.22, 1.67]	0.84 [0.34, 2.10]	0.96 [0.32, 2.84]	1.23 [0.42, 3.57]	0.44 [0.16, 1.19]
d) Business and Industry	1.05 [0.62, 1,77]	0.92 [0.54, 1.57]	1.00 [0.63, 1.59]	1.15 [0.66, 2.00]	0.75 [0.44, 1.28]	1.10 [0.68, 1.79]
e) Environmental Organizations	0.53 [0.19, 1.46]	1.36 [0.50, 3.68]	1.35 [0.56, 3.25]	0.98 [0.34, 2.84]	1.45 [0.54, 3.90]	0.66 [0.27, 1.61]
f) Individuals	1.00 (ref)	1.00 (ref)	1.00 (ref)	1.00 (ref)	1.00 (ref)	1.00 (ref)
g) Other	1.42 [0.66, 3.06]	1.50 [0.68, 3.31]	2.03 [0.97, 4.26]	0.78 [0.34, 1.76]	0.84 [0.37, 1.90]	1.50 [0.70, 3.22]
b vs. all other answers	0.89 [0.67, 1.18]	1.24 [0.94, 1.64]	1.00 [0.78, 1.29]	0.89 [0.66, 1.19]	1.16 [0.88, 1.54]	1.11 [0.85, 1.45]
4. How certain are you that climate change is something that is affecting or going to affect you personally?	−1.55 [−4.93, 1.82]	**6.19 [2.81, 9.57]**^b^	1.67 [−1.38, 4.74]	3.39 [−0.14, 6.93]	0.02 [−3.38, 3.42]	**9.01 [5.80, 12.21]**^b^

Bolded font: *p* <=.001; strongest absolute association per variable is underlined.

a *p* < .05.

b *p <* .001.

**Figure 1:**
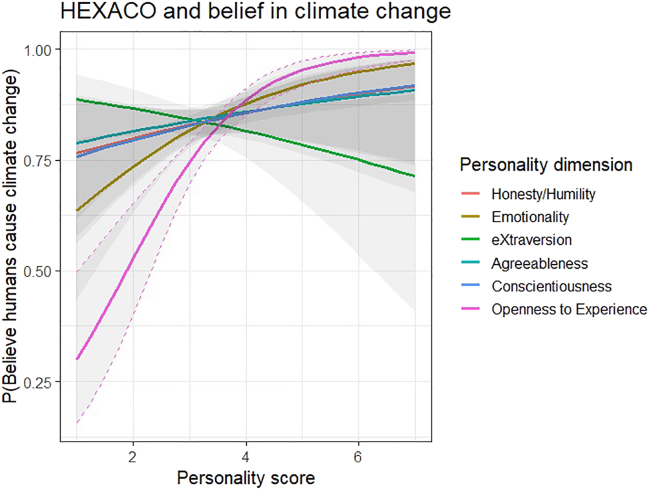
HEXACO and belief in climate change Logistic regression analysis examines the association between HEXACO dimensions and belief in climate change; the dichotomous DV represents belief in climate change, with “1” indicating belief that humans are largely causing climate change, and “0” representing other beliefs. Among the HEXACO dimensions, only Openness had a reliable association with the dichotomous DV (OR = 2.45, Wald = 22.45, *p ≤* 0.001). The grey areas represent 95% confidence intervals.

We analyzed the first DV (“Which of the following best represents your thoughts about climate change?”) with multinomial logistic regression, with “I don’t think that climate change is happening” as the reference category. Only Openness was associated with this DV; the odds of answering “I think that climate change is happening, and I think that humans are largely causing it” (compared with the reference category) increased with increasing scores on Openness (OR = 4.50, 95% CI [1.26, 15.96], *p* < 0.05; see Table 3 for full statistics). However, since the response categories were far from evenly distributed (with only 9 participants in the reference category “I don’t think that climate change is happening”), we reran the analysis by dummy-coding the question: “1 = Humans are largely causing climate change” and “0 = Other.” We then used this dichotomous variable as a DV in a logistic regression analysis with all the HEXACO dimensions simultaneously as predictors. Only Openness was reliably associated (OR = 2.45, Wald = 22.45, *p ≤* 0.001) with the dichotomous DV (see Figure 1).

The second DV (“How certain are you that humans contribute to climate change?”) was analyzed with standard OLS regression. When controlling for the other HEXACO dimensions, only Openness was associated with increased certainty (B = 5.95 [3.29, 8.60], *p* < 0.001). We analyzed the third DV (“Who do you think should have the main responsibility for tackling climate change?”) using multinomial logistic regression with “Individuals” as the reference category. We did not find any associations with the HEXACO dimensions (see Table 3 for full statistics). Finally, the fourth DV (“How certain you are that climate change is something that is affecting or is going to affect you, personally?”) was positively associated with Emotionality (B = 6.19, 95% CI [2.81, 9.57], *p* < 0.001) and Openness (B = 9.01, 95% CI [5.80, 12.21], *p* < 0.001).

In conclusion, Openness was the most important predictor of climate change beliefs (Table 3). Specifically, individuals with high Openness to Experience are more likely to believe that climate change is happening and that humans are causing it, show increased certainty in that belief, and also in the belief that climate change is affecting them or is going to affect them personally.

## Discussion

We examined the relationship between HEXACO personality dimensions and different levels of awareness and connectedness (e.g., Social awareness, Inclusion of Other in Self, Inclusion of Nature in Self), moral orientations related to environmental issues (i.e., Moral awareness, Moral obligation), environmental attitudes (e.g., Concern, Awareness of Consequences), and behaviour (i.e., Ecologically Conscious Consumer Behavior). We found that Openness to Experience is, by far, the most relevant personality dimension in predicting both socio-moral and environmental tendencies. In addition, Openness was by far the most relevant dimension in predicting accurate beliefs about climate change.

More specifically, we found that individuals who are high in Openness were more likely to feel connected to nature, believe that protecting the environment is a moral issue and feel a moral obligation to do so, have environmental views, feel concern, be aware of the negative consequences, believe that climate change is caused by humans, and that they themselves are personally affected. Openness had the most reliable, as well as strong associations (higher B values) with the DVs compared to all the other HEXACO dimensions, in both analyses. In sum, Openness underlies both socio-moral orientations and environmental measures and highlights the connection between them.

Our results are in line with previous research underlining the importance of HEXACO Openness to Experience^32^^,^^33^^,^^34^^,^^36^^,^^37^ and Big Five Openness^31^^,^^33^ for environmentalism. In addition, our findings support previous research showing that Openness aligns to the biospheric value orientations in the Value-Belief-Norm (VBN) model, and it is the primary personality correlate of connectedness.^36^ Past research^39^ has found that people high in Openness have a fuzzier distinction between ingroups and outgroups, and are more likely to not perceive boundaries between humans and other living things; in other words, they are more likely to identify with all humanity and the natural world, thus more likely to protect these aspects.^36^ Similarly, Openness has been argued to “promote attending to and caring about the wider world”^39^; one explanation for this could be that identification with all humanity and moral identity (or an expanding circle of moral regard) are related (ibid.). Our findings highlight the link of Openness to socio-moral orientations such as feeling connected to other people and nature, which contributes to environmental attitudes and behaviors,^6^^,^^11^^,^^14^^,^^18^^,^^19^ thus providing depth in theoretical reasoning regarding the prominence of Openness to Experience in environmental engagement. Moreover, people high in Openness have a high aesthetic appreciation of nature and seek knowledge for it,^40^ which also – from a motivational theory perspective – leads to a tendency to preserve nature.^30^^,^^32^ Soutter et al.^32^ have argued that since Openness is positively correlated with intelligence, knowledge by learning and experience, and being generally informed, it could increase environmentalism through better environmental knowledge and greater awareness of the consequences of human actions on the environment. Our article further supports and broadens these findings in relation to climate change beliefs. Finally, as people high in Openness are, in general, more flexible, unconventional, and often the first ones to adopt new ideas and habits,^40^ they are probably also more willing to engage in environmental behaviors. Regarding climate change, recent findings show that climate change denial is negatively associated with Openness, while proactivity toward climate change is positively associated with Openness.^47^

We further found that individuals high in Emotionality were, in general, more focused on the social aspect of the socio-moral orientations (i.e., awareness of other people, feeling connected to others) as well as feeling concern on all levels. Thus, Emotionality also underlies and connects the socio-moral orientations and the environmental measures (but less so than Openness). In addition, individuals high in Emotionality were likely to believe that climate change is going to affect them personally (Table 3; see also Figure 1). The results are in line with the main characteristics of individuals with high Emotionality who have heightened sensitivity and experience more worry^40^; which has been linked to successfully detecting and dealing with threats.^41^ Furthermore, it seems that high Emotionality may lead to pro-environmental attitudes through empathizing and caring for others, as people high in this dimension are more empathetic, sensitive to other’s feelings, and build strong emotional bonds with other people.^40^ Emotionality has been also previously linked to identification with all humanity, similar to Openness.^39^ Overall, our results give further support to recent findings of a meta-analysis establishing a significant association between HEXACO Emotionality and environmental attitudes, as environmentalism requires an element of empathy for others, animals or the environment.^32^

Similar – but milder – patterns were observed for Extraversion; it seems that through feeling connected to and being aware of others, extraverted individuals are concerned for the environment by focusing more on the social aspect of this issue (e.g., negative consequences for the people). People with high Extraversion are more socially oriented, and it is possible that this is also reflected in their environmental attitudes. In other words, a social orientation may lead extraverted individuals to care about environmental issues mainly in terms of their impact on people. This could be due to the tendency of extraverted individuals to be motivated by experiences and actions that provide a tangible or psychological reward within a social context.^43^^,^^44^ For instance, they may be more likely to engage in group environmental activities where prosocial aspects are emphasized.

Finally, Honesty-Humility was found to be predictive of ecological worldview (i.e., NEP), as well as awareness of negative consequences of environmental degradation (in general, and for one’s self). In addition, it was associated negatively with concern about oneself in the face of environmental degradation. This finding is very revealing in combination with the association of Honesty-Humility with higher awareness of the consequences of environmental degradation for oneself. Taken together, these findings indicate that even highly aware of the egoistic consequences, honest and humble people are not concerned about themselves. Thus, our results support previous findings which link Honesty-Humility to environmental attitudes.^35^^,^^37^ Previous research^36^ has also reported that Honesty-Humility correlates with NEP and environmental behaviors. Honesty-Humility is characterized by modesty, unwillingness to exploit or take advantage of others, as well as little interest in wealth or luxury goods.^40^ Therefore, it can be argued that people high in this dimension can be in alignment with environmentalism through prosocial values.^35^ Finally, our findings are consistent with previous research where Honesty-Humility is associated with moral concern about the welfare of others.^36^

Our article adds to the ongoing discussion, as it examines HEXACO personality dimensions (beyond the traditional Big Five), and combines socio-moral orientations with a wide range of environmental measures (including environmental attitudes, behavior, and climate change beliefs). Although many of the environmental measures have been previously employed in individual studies, here there are multiple measures (socio-moral, environmental attitudes, and so forth) on multiple levels (i.e., individual, social, nature). To our knowledge, there is no overarching analysis of how personality is associated with all the socio-moral orientations combined, and also extensively with a big number of environmental measures.^32^^,^^48^ Our study identifies the personality dimensions that are the common psychological basis for socio-moral orientations and environmental measures, providing a comprehensive overview of the role of personality in environmentalism.

In consistency with previous research, Openness was the most important predictor of environmental attitudes^32^^,^^34^^,^^37^ however, here we also found that it was also the most important predictor of socio-moral orientations. In other words, Openness seems to be a common psychological basis of both these tendencies. This is significant as it explains why Openness should be considered an essential personality dimension for environmentalism. Furthermore, the findings show the unique contribution of Emotionality and Extraversion, as they both also connect environmental measures and socio-moral orientations. In addition, our study gives an overview of how each personality dimension connects socio-moral orientations and environmental tendencies, providing a better understanding of why some personality dimensions are more prominent than others in environmental engagement, thus advancing our current knowledge of the role of personality in environmentalism. Finally, our article makes a unique contribution to the literature by examining HEXACO dimensions in conjunction with climate change beliefs and showing that Openness is associated with such beliefs as well.

Our results highlight the importance of Openness to Experience in socio-moral orientations and environmental measures, and align with prior research on HEXACO personality dimensions and environmental attitudes. When the two samples were analyzed separately, the prominence of Openness to Experience was observed in both of them. Therefore, we expect this finding to generalize to other populations as well. While we expect some variations due to cultural differences, we believe that the prominence of Openness to Experience will be reproducible among different populations. A direct replication would test HEXACO dimensions in conjunction with socio-moral orientations and environmental measures in other samples. We have no reason to believe that the results depend on other characteristics of the participants, materials, or context.

Future research is needed to replicate these results in other samples. Socio-moral orientations such as interdependence of life, and moral awareness of environmental issues should be examined more deeply in relation to environmentalism. For instance, feeling interconnected to other people may be a crucial factor in pro-environmental action that should not be overlooked. As the environmental issue becomes more and more urgent, socio-moral orientations could address some of the gaps in our current knowledge. In addition, future studies could explore which specific aspects of Openness, such as aesthetic appreciation, are more associated with environmental attitudes and socio-moral orientations than other sub-facets of Openness. This could help to better understand the nuances of personality that are associated with environmentalism. Finally, future research could look into the mediation effects of socio-moral orientations on environmental measures.

The findings of our study underscore several key practical implications for addressing the environmental challenge through the lens of personality and designing interventions aimed at promoting environmentalism. Policymakers could design initiatives that consider the diverse personality profiles to engage a broader audience. For instance, highlighting moral aspects may resonate with individuals who prioritise ethical considerations – such as individuals high in Honesty-Humility – while emphasizing personal and communal benefits might attract those motivated by social and practical concerns – such as emotional and extraverted individuals. Similarly, environmental campaigns could be designed to appeal to specific personality traits to achieve a bigger impact. As individuals high in Openness to Experience are more likely to be aware of ecological issues and adopt pro-environmental attitudes, environmental campaigns can leverage this trait by promoting content that resonates with these individuals. In addition, environmental protection campaigns could target extraverted individuals by underlining how natural settings could provide meaningful social experiences and societal advantages, or individuals with high sensitivity by focusing on the dangers of environmental degradation for humans and other species. Finally, educational programs aiming at raising ecological awareness could cultivate a strong environmental ethic by highlighting the connection of people to nature and to humanity and foster positive attitudinal and behavioral changes. By acknowledging and leveraging the diversity in personality traits, more inclusive and impactful strategies can be developed to encourage a widespread environmental consciousness and action.

Our article highlights the importance of personality dimensions in environmental research and illustrates the prominence of Openness to Experience in raising ecological awareness and adopting environmental attitudes, as well as in establishing accurate climate change beliefs. It is possible that in the future, people who are high in this dimension will drive the change in becoming more environmentally conscious and adopting more ecological manners. Most crucially, however, this article highlights that in the future, if humanity is to tackle climate change, we need to understand how to reach people with all sorts of personalities and not just those who are highly open, since they consist only of a small minority. In addition, our article includes socio-moral orientations that have not been investigated thoroughly regarding environmental issues, showing possible future research directions in understanding and addressing climate change.

### Limitations of the study

Our study has the usual limitations of cross-sectional social psychological studies. We cannot infer causality, neither we can establish the direction of the relationships. Another limitation is potential common method bias, which is typical in psychology, as both the IVs and the DVs were measured with self-report Likert scales within the same survey session. It is possible that the observed associations could be inflated due to factors such as response consistency. In addition, the laboratory sample consisted mostly of students (Finnish sample), while the online participants were probably people educated and technologically savvy with computer literacy (English-speaking sample), making both of the samples non-representative. In addition, all of our measures were self-report ones related to well-known weaknesses; for instance, the self-report evaluation of environmental behavior may include some discrepancy between real behaviors and reported behaviors. However, we tried to mitigate these limitations by combining the data to examine the robustness of our results. We focused mainly on the highly significant results (*p* ≤ 0.001) not allowing for the overinterpretation of weak signals or false positives. Furthermore, when performing multiple regression analysis, there could be potential bias in the B-estimates due to multicollinearity. However, assessing the degree of multicollinearity by examining the VIF values showed that there is no high multicollinearity (all VIF values ≤1.26); also, since the HEXACO dimensions do not share more than about 6% of variance, they are, for all practical purposes, orthogonal. Another potential limitation is the conceptual overlap and thus the correlation of the DVs. In order to address this potential confound, we looked at the B-values with both multiple regression and path analysis methods (in other words, allowing cross-correlations between the DVs produced identical results to those reported here; we present the simplest analyses, following the principle of parsimony). Regarding the measures, we did not use the whole ECCB scale, and we also created our own item of moral obligation, which has not been excessively validated. Also, the first climate change belief item was dichotomized due to thin categories, which limits interpretability. Finally, the study was not preregistered although preregistration comes with many benefits, and future studies will follow this practice.

## Resource availability

### Lead contact

Further information can be obtained from the lead contact: Marianna Drosinou, email: maria-anna.drosinou@helsinki.fi.

### Materials availability

Only a single item of Moral Obligation was generated for this study. The item can be found in the materials section of the STAR Methods.

### Data and code availability

• The dataset generated and analyzed during the current study is available as of the date of publication on figshare.com doi: https://doi.org/10.6084/m9.figshare.27764931.
• The code used for statistical analysis is available as of the date of publication on figshare.com doi: https://doi.org/10.6084/m9.figshare.30138829.
• There are no other items.

## Acknowledgments

This project would not have been possible without a generous grant from the 10.13039/501100019787Tiina and Antti Herlin Foundation to study climate change (www.tah.fi). We would also like to give a warm thanks to Moralities of Intelligent Machines (www.moim.fi) for all the valuable help during this project.

## Author contributions

Marianna Drosinou: conceptualization, methodology, software, formal analysis, investigation, resources, data curation, writing – original draft, writing – review and editing, project administration, and funding acquisition. Jussi Palomäki: software, formal analysis, resources, writing – review and editing, visualization, and supervision. Anton Kunnari: data collection and software. Mika Koverola: data collection. Markus Jokela: writing – review and editing and supervision. Michael Laakasuo: software, validation, formal analysis, resources, writing – original draft, writing – review and editing, visualization, and supervision.

## Declaration of interests

The authors declare no competing interests.

## STAR★Methods

### Key resources table

**Table:** 

REAGENT or RESOURCE	SOURCE	IDENTIFIER
**Deposited data**		

Hexaco and Environmentalism	Figshare	https://doi.org/10.6084/m9.figshare.27764931

**Software**		

SPSS 28	IBM	https://www.ibm.com/products/spss
R Studio	R Project	https://www.r-project.org/

### Experimental model and study participant details

#### Participants

First, 236 (*N* = 236; 129 female) participants (Age_M_ = 31.68; SD; = 11.40; Range = 18–77) were recruited in the Central Library of the University of Helsinki to participate in a 20–30 min study. Out of the total number of participants, 224 (94.9%) resided in Finland, 132 (55.93%) had at least a Bachelor’s degree, 131 (55.5%) were students, 110 (46.6%) were single, 188 (79.7%) had no children, 135 (57.2%) had an annual income between 0€ and 19.000€, and 64 (27.11%) excluded meat from their diets.

In addition, 398 (*N* = 398; 211 female) participants (Age_M_ = 32.92; SD; = 11.36; Range = 18–75) were recruited online on Prolific (www.prolific.co). Out of the total number of participants, 206 (51,7%) resided in the UK (while the rest resided in other English-speaking countries), 204 (51.6%) had at least a Bachelor’s degree, 190 (48.2%) were waged employed, 147 (36.9%) were single, 227 (57.6%) had no children, 146 (37.1%) had an annual income between 20.000 and 39.999₤, and 50 (12.6%) said they exclude meat from their diets.

The requirement of ethical approval is waived by University of Helsinki Ethical Review Board in Humanities and Social and Behavioral sciences for the studies involving humans, as according to the guidelines of the Finnish National Board on Research Integrity, this type of research does not require ethical review (https://www.tenk.fi/en). The studies were conducted in accordance with the local legislation and institutional requirements.

### Method details

#### Ethics statement

All local laws governing research ethics were complied with in full. All APA ethical guidelines were followed. The research procedures and protocols were covered by the ethics statement by the University of Helsinki Ethical Review Board in Humanities and Social Sciences.

#### Procedure

The laboratory data were gathered in our pop-up laboratory, in a large public library in the Helsinki Metropolitan Area. Participant recruitment was non-intrusive: our research assistants, dressed in neutral clothing, were sitting behind a desk with the sign “Participate in Psychological Research”, in the foyer of the library. All recruited participants approached our research assistants voluntarily. Participants first confirmed that they were over 18-years old, and then gave their informed consent to participate in a study on “how personal preferences and beliefs are associated with lifestyle choices”. After completing the online questionnaire, participants were debriefed, paid, and thanked for their time.

For the online data, participants first gave their informed consent to participate in a study on “how personal preferences and beliefs are associated with lifestyle choices”. After completing our online questionnaire, participants were debriefed, and thanked for their time. Compensation was according to the requirements of Prolific on ethical rewards.

In both samples, we used software that prevents duplicate answers. In addition, we checked for duplicates and there were none.

#### Design

We designed a cross-sectional study to explore the associations between personality dimensions and several levels of connectedness/awareness, environmental attitudes and behaviors. As independent variables, we used the HEXACO personality dimensions, and as dependent variables we employed a variety of socio-moral orientations (e.g., self- and other-awareness, as well as moral awareness and moral obligation) and environmental measures (e.g., environmental concern, environmental views, self-reported consumer behavior). For more details, see below and Table S4; for the correlations between the dependent variables, see Table S5.

#### Materials

##### Independent variable

###### HEXACO-60 personality inventory

We used the HEXACO-PI-R (also known as HEXACO-60)^27^ to assess personality. HEXACO is a six-dimensional instrument with very good psychometric properties in personality assessment.^49^^,^^50^ It is similar to the Big Five or Five Factor Model constructs, with the exception of the 6^th^ dimension labeled “Honesty-Humility”. In HEXACO-60, each subscale consists of 10 items; all items were anchored from 1 = *Strongly Disagree* to 5 = *Strongly Agree*. Honesty-Humility reflects unwillingness to manipulate others for personal gain, a low tendency to break rules, and lack of interest in status symbols or a luxurious lifestyle (α = 0.70). Emotionality measures the tendency to be sentimental, experience fear, anxiety, and empathy, as well as a need of emotional support (α = 0.73); Extraversion reflects a tendency to feel positive about oneself, be confident in social gatherings, and experience feelings of enthusiasm and energy (α = 0.80); Agreeableness measures the tendency to be forgiving, patient, flexible, and have leniency in one’s judgments (α = 0.71); Conscientiousness measures the tendency to stay organised, work hard, be disciplined, and strive for perfection (α = 0.73); and Openness to Experience (α = 0.77) measures the tendency to have a high aesthetic appreciation of art and nature, be creative, imaginative, inquisitive, and unconventional (for further details, see Lee and Ashton^40^).

##### Dependent variables

###### Mindful attention awareness scale (MAAS)

Mindful attention to the present moment was measured with the MAAS.^51^ All items were reversed coded (e.g., “I could be experiencing some emotion and not be conscious of it until some time later.”, “I find it difficult to stay focused on what’s happening in the present.”) so that higher scores reflect higher levels of dispositional mindfulness. Items were anchored from 1 = *Almost Never* to 7 = *Almost Always* (α = 0.87).

###### Social awareness inventory (SAI)

To measure self- and other-awareness we used two factors from the SAI.^52^ The first factor *Self experience from the self perspective* (hereafter Self-awareness) includes 7 items (e.g., “I reflect about myself and my inner motives a lot.”, “To help myself become the person I want to be, I frequently reassess my reactions to things.”), while the sixth factor *Other’s experience from the other’s perspective* (hereafter Other-awareness) includes 8 items (e.g., “When talking to others I tend to get absorbed in their concerns, even if they are not my concerns.”, “I find it natural to identify with other’s needs.”). All items were anchored from 1 = *Not at all like me* to 5 = *Very much like me*. Higher scores reflect higher levels of self-awareness (α = 0.87) and other-awareness (α = 0.90) respectively.

###### Inclusion of Other in Self (IOS)

Using seven pictures of overlapping circles representing *Self* and *Other,* participants were asked to choose the number of the picture that best showed how interconnected they feel to other people in general.^15^ Scores ranged from 1 (where the circles touched but did not overlap) to 7 (where the circles almost completely overlapped); higher scores show higher levels of interconnectedness with others.

###### Inclusion of Nature in Self (INS)

Similarly to IOS, using a series of overlapping circles representing *Self* and *Nature,* participants were asked to choose the number of the picture that best showed how interconnected they feel to nature in general.^7^^,^^10^ Scores ranged from 1 (where the circles touched but did not overlap) to 7 (where the circles were almost completely overlapping); higher scores show higher levels of interconnectedness with nature.

###### Moral Awareness

We measured being morally aware of the environmental protection issue (i.e., perceiving that it contains moral content) with two items adapted for this study from Reynolds^53^: *“*There are very important ethical aspects to environmental protection*”* and “Protecting the environment could be described as a moral issue”. The items were evaluated on a scale from 1 = S*trongly Disagree* to 7 = S*trongly Agree* (α = 0.76).

###### Moral Obligation

We measured moral obligation toward the environment with a single item “I feel a moral obligation to protect the environment”. Previously, similar single items have been used to measure a moral duty to react to climate change^29^ and to protect the environment by consuming fewer resources.^54^ Participants rated the item on a 5-point scale, ranging from *Strongly Disagree* to *Strongly Agree*.

###### Environmental self-identity

Three items (α = 0.90) were used to measure whether participants perceived themselves as environmentally friendly: “Acting environmentally friendly is an important part of who I am”; “I am the type of person who acts environmentally friendly”; “I see myself as an environmental-friendly person”.^3^ All items were anchored from 1 = *Strongly Disagree* to 5 = *Strongly Agree*.

###### Environmental Viewpoints

Views regarding the natural environment were measured with 5 items developed by Ellis and Thompson^55^ and adjusted by Dutcher et al.^9^ Items include “We are fast using up the world’s resources.”, and “If things continue on their present course, we will soon experience a major ecological catastrophe”. All items were anchored from 1 = *Strongly Disagree* to 7 = *Strongly Agree*. Two items were reversed coded so that higher scores reflect more environmental views (α = 0.80).

###### Environmental concern

Concern for the environmental problems was measured with 12 items.^10^ Participants were asked to rate the items in response to the question: I am concerned about environmental problems because of the consequences for: “Plants and trees”, “Marine life”, “Birds and Animals” (*Biospheric concern*, α = 0.93); *“*Humanity”, “People in my Community”, “Future generations” (*Social* concern*,* α = 0.85); “My future”, “My health”, “My lifestyle” (*Egoistic concern,* α = 0.81). All items were anchored from 1 = *Not at all important* to 7 = *Very important*. A score was computed for each subscale separately, as well as for all the 12 items (hereafter General Concern). Higher scores reflect higher biospheric, social and egoistic concern respectively.

###### Short new ecological paradigm (short NEP)

To measure individual differences in environmental worldview, we used the 6-item shortened version^2^ of the revised NEP^56^; three items reflect seeing the environment as a commodity to be used by humans, and the other three reflect seeing it as valuable on its own and in need of protection. Items include “Nature is strong enough to cope with the impact of modern industrial nations.” and “The balance of nature is very delicate and easily upset.” (α = 0.74). The items were evaluated on a 7-point scale (1 = *Strongly Disagree*; 7 = *Strongly Agree*). Three items were reversed coded so that higher scores reflect a higher ecocentric orientation, namely a higher commitment to preserve the natural resources.^57^

###### Awareness of Consequences scale (AC)

The AC scale^58^ measures the degree to which people are aware of the environmental consequences regarding themselves, other people, and nature. AC consists of 15 items (α = 0.89) that are further categorised into three 5-item subscales: *Egoistic* (e.g., “Environmental protection is beneficial to my health.”, “Environmental protection will provide a better world for me and my children.”; two items were reversed coded; α = 0.66), *Social* (e.g., “Environmental protection benefits everyone.”, “Pollution generated here, harms people all over the Earth.”; one item was reversed coded; α = 0.79), and *Biospheric* (e.g., “Over the next several decades, thousands of species will become extinct.”, “Modern development threatens wildlife.”; two items were reversed coded; α = 0.76). Items were measured on a 7-point scale from *Strongly Disagree* to *Strongly Agree*. A score was computed for each subscale separately, as well as for all the 15 items (hereafter General Awareness of Consequences). Higher scores reflect higher awareness of egoistic, social and biospheric consequences respectively.

###### Ecologically conscious consumer behavior (ECCB)

ECCB consists of 30 items in total, categorised into six factors, each having a focus on a specific environmental behavior (e.g., recycling). For the purposes of our study, we used the fourth factor from the ECCB, which measures ecological buying decisions with 12 items; items include: “I normally make a conscious effort to limit my products that are made of or use scarce resources.”, “I have switched products for ecological reasons.”, “When I have a choice between two equal products, I always purchase the one which is less harmful to other people and the environment.*”* (α = 0.93). Items were anchored from 1 = *Never true* to 7 = *Always true*; one item was reversed coded so that higher scores reflect more ecologically conscious buying behavior.^59^

###### Climate change beliefs

Participants’ climate change beliefs and environmental biases were assessed with four different measures. First, we measured climate change beliefs with a single nominal item asking participants to answer the following question: “Which of the following best represents your thoughts about climate change? a) Ι don’t think that climate change is happening, b) I have no idea whether climate change is happening or not, c) Climate change is happening, but it’s just a natural fluctuation, and d) Climate change is happening, and humans are largely causing it.”.^28^ Second, participants were asked to indicate how certain they were that humans are causing climate change, on a slider ranging from 0 to 100.^54^ Third, participants were asked to indicate who has the main responsibility for tackling climate change among the following options: “National government; Local government; Business and industry; International organisations; Environmental organisations/lobby groups; Individuals; Other”.^29^ Finally, we measured perceived risk with a single item: “How certain you are that climate change is something that is affecting you or is going to affect you personally” adjusted from Whitmarsh.^29^ Participants gave their scores on a slider ranging from 0 to 100.

### Quantification and statistical analysis

We first analyzed the two samples separately and the results were similar, thus we decided to combine the datasets to increase statistical power and improve the robustness of our findings (for results and comparison between the two samples, see Tables S6 and S7). We employed ordinary least squares (OLS) regression and regressed each HEXACO dimension individually on each of our DVs, controlling for nationality, age, sex, and education (for the simple regression analysis, see Table 1). We then ran OLS multiple regression analyses with all HEXACO dimensions as predictors simultaneously, thus controlling for their effects - as well as the effects of the control variables -, and regressed them on each of our DVs. The results of the controlled (multiple) regression analyses are shown in Table 2; all associations below the *p* ≤ 0.001 -level have been bolded, and the strongest association per variable has been underlined. The results are presented in order of HEXACO dimension prominence.

Since there were 19 DVs (when counting each subscale as a separate DV), we apply the Bonferroni correction -method and primarily focus only on the associations that are significant at the *p ≤* 0.001 -level (we refer to these as “reliable” associations) (Since there were 19 variables, following the Bonferroni method, we should divide 0.05 by 19 (using the conventional p-level), which results in 0.002. However, to err on the side of caution, we use a more conservative p-level of 0.001, which further avoids the potential for false positives). Given the large number of variables being studied, to guard against type-1 errors, we strictly interpret *p ≤* 0.001 as *significant*, and *p* > 0.001 as *non-significant.*

In all statistical models, the model residuals were homoscedastic and normally distributed based on visual inspection of Q-Q plots, and the association between the variables was linear as per the assumptions of general linear models. See Figure S1 for a scatterplot matrix of all HEXACO dimensions, Figure S2 for a histogram panel figure of all DVs, and Figure S3 for their multiple regression residuals. In all of the multiple regression analyses, multicollinearity diagnostics were evaluated. In summary, there was no multicollinearity between the independent variables; all variance inflation factor (VIF) values ≤1.26.

In our most complicated models with 10 predictors, assuming a conservative effect size (of 3% of explained variance), about 600 individuals are needed for a power of about 0.85. All our analyses included more than 600 individuals. Omitting control variables such as nationality, sex, age, and education would yield a statistical power of 0.9 to detect a small effect size (3% of variance explained) with a sample size of about 560. Thus, our sample size, based on very conservative estimations, is large enough to answer our research questions (https://www.statskingdom.com/33test_power_regression.html).

#### Additional resources

This study did not utilize or generate additional tools, software, or databases beyond standard survey platforms and statistical software.
