# Supplementary material for: HEXACO personality dimensions as predictors of environmental attitudes, socio-moral orientations, and climate change beliefs
Source: iScience. 2025 Oct 17;28(11):113753. doi: 10.1016/j.isci.2025.113753 (PMC12616036; doi:10.1016/j.isci.2025.113753)
Supplement: Document S1. Figures S1–S3 and Tables S1–S7 [file mmc1.pdf]

iScience, Volume *28*

Supplemental information

HEXACO personality dimensions as predictors of environmental attitudes, socio-moral orientations, and climate change beliefs

Marianna Drosinou, Jussi Palomäki, Anton Kunnari, Mika Koverola, Markus Jokela, and Michael Laakasuo

Data S1/Methods S1. Tables S1 - S7 and Figures S1 - S3

HEXACO personality dimensions as predictors of environmental attitudes, socio-moral orientations, and climate change beliefs

Marianna Drosinou [a, b, *,**], Jussi Palomäki [c, d], Anton Kunnari [a, d], Mika Koverola [d], Markus Jokela[a], Michael Laakasuo [d, e ***]

[a] University of Helsinki, Faculty of Medicine, Discipline of Psychology, Helsinki, Finland

[b] University of Turku, Faculty of Social Sciences, Department of Psychology, Turku, Finland

[c] Finnish Institute for Health and Welfare, Department of Public Health and Welfare, Helsinki, Finland

[d] University of Helsinki, Faculty of Arts, Cognitive Science Unit, Helsinki, Finland

[e] University of Turku, Faculty of Social Sciences, Department of Social Research, Turku, Finland

[*] Corresponding author: maria-anna.drosinou@helsinki.fi

[**] Lead contact

[***] Principal Investigator, Senior Author

R^2 in Simple direct regression associations

Table S.1: R^2 in Simple direct regression associations between HEXACO dimensions and dependent variables (N = 634)

Predictors	Mindful Attention Awareness	Social Awareness of Self	Social Awareness of Other	Inclusion of Other in Self	Inclusion of Nature in Self	Moral Awareness	Moral Obligation	Environmental Self-identity	Environmental Viewpoints	General Concern	Biospheric Concern	Social Concern	Egoistic Concern	New Ecological Paradigm	Awareness of Consequences -General	Awareness of Consequences - Biospheric	Awareness of Consequences - Social	Awareness of Consequences - Egoistic	Eco-conscious Consumer Behaviour
Honesty-Humility	.05	.05	.03	.01	.03	.11	.10	.06	.10	.02	.07	.06	.05	.11	.12	.13	.06	.14	.06
Emotionality	.03	.07	.11	.02	.03	.11	.07	.04	.09	.06	.08	.07	.02	.08	.09	.10	.03	.10	.04
Extraversion	.04	.05	.05	.19	.05	.09	.09	.06	.08	.05	.04	.09	.02	.07	.08	.10	.03	.09	.06
Agreeableness	.03	.05	.07	.02	.03	.10	.08	.07	.08	.03	.05	.07	.01	.07	.09	.10	.03	.09	.07
Conscientiousness	.14	.06	.03	.01	.03	.09	.08	.04	.09	.02	.04	.04	.01	.07	.10	.11	.04	.12	.05
Openness	.05	.13	.06	.02	.07	.15	.16	.10	.16	.04	.11	.07	.01	.13	.21	.20	.11	.21	.10

Notes: the analyses have been controlled for demographic variables

Semi-partial correlations

Table S.2: Semi-partial correlations between HEXACO dimensions and dependent variables (N = 634)

Predictors	Mindful Attention Awareness	Social Awareness of Self	Social Awareness of Other	Inclusion of Other in Self	Inclusion of Nature in Self	Moral Awareness	Moral Obligation	Environmental Self-identity	Environmental Viewpoints	General Concern	Biospheric Concern	Social Concern	Egoistic Concern	New Ecological Paradigm	Awareness of Consequences -General	Awareness of Consequences - Biospheric	Awareness of Consequences - Social	Awareness of Consequences - Egoistic	Eco-conscious Consumer Behaviour
Honesty-Humility	.14	-.14	-.00	.01	.04	.07	.10	.07	.07	-.01	.08	.09	-.18	.13	.12	.10	.09	.14	.07
Emotionality	-.10	.19	.34	.21	.02	.13	.06	.07	.09	.24	.20	.23	.13	.09	.08	.03	.10	.10	.04
Extraversion	.07	.03	.19	.43	.11	.05	.08	.13	-.04	.18	.05	.24	.13	-.02	-.00	-.06	.01	.04	.08
Agreeableness	.03	.05	.19	.06	.03	.02	.04	.10	.03	.06	.04	.10	-.00	.01	.01	.00	.02	-.00	.09
Conscientiousness	.28	.09	-.08	-.02	.01	-.01	.00	-.00	.05	-.03	-.01	-.04	-.02	.01	.08	.05	.07	.09	-.00
Openness	.09	.28	.16	.04	.17	.21	.26	.21	.26	.14	.23	.13	-.02	.21	.31	.29	.25	.29	.20
Nationality	-.02	-.07	.07	.13	.12	-.15	-.11	-.04	-.13	.02	-.00	-.02	.08	-.06	-.10	-.16	-.01	-.11	.00

Notes: the analyses have been controlled for demographic variables

Climate Change Beliefs items and statistics

Table S.3: Climate Change Beliefs statistics (N = 634)					
**Climate Change Beliefs:**	**Frequency**	**Percent %**	**Cum. Percent %**	**Mean**	**SD**
**1. Which of the following best represents your thoughts about climate change?**					
- I don't think that climate change is happening.	8	1.3	1.3		
- I have no idea whether climate change is happening or not.	47	7.5	8.8		
- I think that climate change is happening, but it's just a natural fluctuation in Earth's temperatures.	60	9.6	18.4		
- I think that climate change is happening, and I think that humans are largely causing it.	511	81.6	100		
**2. How certain are you that humans contribute to climate change?**				80.71	21.38
**3. Who do you think should have the main responsibility for tackling climate change?**					
National government	276	44.1	44.1		
Local government	14	2.2	46.3		
Business and Industry	133	21.2	67.5		
International organisations	90	14.4	81.9		
Environmental organisation/ lobby groups	16	2.6	84.5		
Individuals	68	10.9	95.4		
Other	29	4.6	100		
**4. How certain are you that climate change is something that is affecting or going to affect you personally?**				68.13	25.69

Descriptive statistics

**Table S.4: Variables and statistics (N = 634)**					
**Scale**	**Mean**	**SD**	**Items**	**Range**	**Cronbach's α**
**HEXACO:**			60		
Honesty-Humility	3.37	.64	10	1-5	.70
Emotionality	3.34	.63	10	1-5	.73
Extraversion	3.09	.69	10	1-5	.80
Agreeableness	3.10	.59	10	1-5	.71
Conscientiousness	3.40	.60	10	1-5	.73
Openness to Experience	3.60	.67	10	1-5	.77
**Environmental Self Identity**	3.53	.93	3	1-5	.90
**MAAS**	4.19	1.03	15	1-7	.87
**SAI:**			15		
Self Awareness	3.65	.81	7	1-5	.87
Other Awareness	3.30	.85	8	1-5	.90
**IOS**	3.76	1.52	1	1-7	-
**INS**	3.95	1.67	1	1-7	-
**Moral Awareness**	5.61	1.25	2	1-7	.76
**Moral Obligation**	3.71	1.06	1	1-5	-
**Environmental Viewpoints**	5.55	1.19	5	1-7	.80
**Environmental Concern:**	5.43	1.02	12	1-7	
Biospheric	5.73	1.30	4	1-7	.93
Social	5.72	1.28	4	1-7	.85
Egoistic	4.83	1.39	4	1-7	.81
**NEP (short)**	5.24	1.08	6	1-7	.74
**Awareness of Consequences:**	5.64	.93	15	1-7	
Biospheric	5.67	1.05	5	1-7	.76
Social	5.68	1.04	5	1-7	.79
Egoistic	5.56	.97	5	1-7	.66
**ECCB (fourth factor)**	4.38	1.35	11	1-7	.93

Correlations

Table S.5. Correlations between dependent variables (N = 634)

	Mindful Attention Awareness	Social Awareness of Self	Social Awareness of Other	Inclusion of Other in Self	Inclusion of Nature in Self	Moral Awareness	Moral Obligation	Environmental Self-identity	Environmental Viewpoints	General Concern	Biospheric Concern	Social Concern	Egoistic Concern	New Ecological Paradigm	Awareness of Consequences -General	Awareness of Consequences - Biospheric	Awareness of Consequences - Social	Awareness of Consequences - Egoistic	Eco-conscious Consumer Behaviour
Mindful Attention Awareness																			
Social Awareness of Self	-.128**																		
Social Awareness of Other	-.145**	.402**																	
Inclusion of Other in Self	.088*	.145**	.330**																
Inclusion of Nature in Self	.106**	.084*	.152**	.285**															
Moral Awareness	-0.048	.233**	.234**	.093*	.184**														
Moral Obligation	0.011	.194**	.189**	.094*	.283**	.509**													
Environmental Self-identity	0.005	.167**	.248**	.147**	.377**	.370**	.716**												
Environmental Viewpoints	.131**	0.058	.153**	0.054	.238**	.395**	.403**	.372**											
General Concern	0.072	.144**	.136**	0.065	.158**	.445**	.471**	.388**	.705**										
Biospheric Concern	-0.049	.254**	.310**	.213**	.272**	.361**	.382**	.382**	.253**	.340**									
Social Concern	-0.025	.226**	.308**	.112**	.316**	.461**	.474**	.442**	.476**	.463**	.772**								
Egoistic Concern	-0.004	.203**	.295**	.271**	.204**	.343**	.347**	.332**	.209**	.303**	.836**	.569**							
New Ecological Paradigm	-.082*	.164**	.126**	.118**	.118**	0.050	.080*	.125**	-.079*	0.038	.721**	.248**	.396**						
Awareness of Consequences -General	.156**	.151**	.140**	.109**	.197**	.572**	.478**	.426**	.713**	.748**	.363**	.509**	.320**	0.032					
Awareness of Consequences - Biospheric	.207**	.171**	.135**	.140**	.193**	.494**	.418**	.398**	.622**	.615**	.323**	.438**	.314**	0.015	.901**				
Awareness of Consequences - Social	0.067	.141**	.169**	.137**	.248**	.573**	.472**	.430**	.640**	.681**	.403**	.508**	.340**	.103**	.916**	.751**			
Awareness of Consequences - Egoistic	.154**	.101*	.078*	0.023	.097*	.488**	.410**	.334**	.678**	.735**	.262**	.437**	.220**	-0.032	.905**	.715**	.739**		
Eco-conscious Consumer Behaviour	0.004	.144**	.257**	.154**	.401**	.405**	.569**	.672**	.365**	.373**	.428**	.516**	.357**	.134**	.442**	.391**	.484**	.328**	

Notes: *: p <.05, **: p < .01, ***: p <= .001; Bolded font: p <=.001;

Interpretation of Finnish Sample

When regression analysis was run by controlling for the effects of other personality dimensions, Openness was found to be prominently implicated in all analyses. Openness was found to have reliable associations with 10 DVs (all Bs > 0.31, $p \leq .001$), including both socio-moral orientations (e.g. Self-awareness, Moral awareness, Moral obligation), and pro-environmental measures (e.g. General Concern, General Awareness of Consequences). It also had the strongest associations (i.e. highest B-values) with 11 DVs compared to the other dimensions. These results highlight the importance of Openness in being socially and morally orientated as well as in having pro-environmental attitudes and beliefs. In other words, Openness was found to underlie both the socio-moral orientations and the environmental attitudes.

Emotionality was found to have reliable associations (all Bs > 0.25, $p \leq .001$) with 8 DVs. It had the strongest associations with Other-Awareness, General Concern, and NEP.

Extraversion had reliable associations with 5 DVs (all Bs > 0.24, $p \leq .001$) including IOS, Social Concern, Awareness of Consequences (Social and Egoistic). It was also found to have the strongest association with IOS, and Awareness of Consequences – Social. Thus, Extraversion seems to be related to pro-environmental measures through its focus on the social aspect.

Honesty-Humility was found to have reliable associations with 3 DVs (all Bs > 0.30, $p \leq .001$). Honesty-Humility had the strongest associations with Egoistic Concern, Awareness of Consequences – Egoistic, and ECCB; where the association with the Egoistic Concern was negative. In other words, environmental concern for one's self was reduced with increased Honesty-Humility, indicating that although people with high Honesty-Humility are concerned about nature in general, they are not concerned about themselves. In addition, Honesty-Humility was the most important dimension for pro-environmental behaviour (i.e. ECCB).

Conscientiousness was only found to have an unsurprising idiosyncratic association (B = 0.43, $p \leq .001$) with MAAS, while that was also the strongest association for this DV. Finally, Agreeableness was not found to have any reliable associations (at $p \leq .001$ -level) with the DVs.

Table S.6. Multiple regression associations between HEXACO dimensions and dependent variables (Finnish sample, N = 236)

	Mindful Attention Awareness	Social Awareness of Self	Social Awareness of Other	Inclusion of Other in Self	Inclusion of Nature in Self	Moral Awareness	Moral Obligation	Environmental Self-identity	Environmental Viewpoints	General Concern	Biospheric Concern	Social Concern	Egoistic Concern	New Ecological Paradigm	Awareness of Consequences - General	Awareness of Consequences - Biospheric	Awareness of Consequences - Social	Awareness of Consequences - Egoistic	Eco-conscious Consumer Behaviour
	B [95% CI]	B [95% CI]	B [95% CI]	B [95% CI]	B [95% CI]	B [95% CI]	B [95% CI]	B [95% CI]	B [95% CI]	B [95% CI]	B [95% CI]	B [95% CI]	B [95% CI]	B [95% CI]	B [95% CI]	B [95% CI]	B [95% CI]	B [95% CI]	B [95% CI]
Predictors																			
Honesty-Humility	0.30 [0.11, 0.49]**	-0.21 [-0.36, -0.06]**	-0.04 [-0.19, 0.10]	0.06 [-0.19, 0.32]	0.08 [-0.24, 0.41]	0.13 [-0.08, 0.34]	0.13 [-0.05, 0.32]	0.16 [-0.01, 0.34]	0.26 [0.04, 0.47]*	0.05 [-0.13, 0.25]	0.36 [0.13, 0.60]**	0.29 [0.07, 0.51]*	**-0.48 [-0.76, -0.20]*****	0.28 [0.09, 0.47]**	**0.30 [0.16, 0.44]*****	0.23 [0.05, 0.40]*	0.26 [0.08, 0.43]**	**0.41 [0.26, 0.55]*****	0.33 [0.08, 0.59]**
Emotionality	-0.17 [-0.35, 0.00]	0.16 [0.02, 0.31]*	**0.58 [0.43, 0.72]*****	**0.47 [0.22, 0.72]*****	0.09 [-0.21, 0.40]	**0.38 [0.17, 0.58]*****	0.18 [-0.00, 0.37]*	0.13 [-0.04, 0.30]	0.31 [0.10, 0.51]**	**0.42 [0.23, 0.60]*****	**0.49 [0.27, 0.72]*****	0.32 [0.11, 0.53]**	**0.44 [0.17, 0.71]*****	**0.30 [0.11, 0.48]*****	0.19 [0.06, 0.33]**	0.11 [-0.05, 0.28]	0.21 [0.05, 0.38]*	**0.25 [0.12, 0.39]*****	0.01 [-0.23, 0.25]
Extraversion	0.22 [0.02 0.42]*	-0.03 [-0.18, 0.12]	0.19 [0.03, 0.34]*	**1.01 [0.73, 1.27]*****	0.33 [-0.00, 0.67]	0.12 [-0.10, 0.34]	0.13 [-0.06, 0.33]	0.12 [-0.06, 0.31]	0.08 [-0.13, 0.30]	0.30 [0.09, 0.50]**	0.24 [-0.00, 0.48]*	**0.48 [0.25, 0.71]*****	0.16 [-0.12, 0.46]	0.18 [-0.01, 0.38]	**0.24 [0.09, 0.38]*****	0.10 [-0.07, 0.29]	**0.30 [0.11, 0.48]*****	**0.31 [0.15, 0.46]*****	0.23 [-0.03, 0.49]
Agreeableness	-0.01 [-0.21, 0.18]	0.03 [-0.12, 0.19]	0.21 [0.05, 0.37]*	0.12 [-0.14, 0.40]	-0.07 [-0.42, 0.27]	0.10 [-0.12, 0.33]	0.04 [-0.16, 0.24]	0.19 [0.00, 0.38]*	0.11 [-0.11, 0.34]	-0.02 [-0.23, 0.17]	-0.02 [-0.27, 0.22]	0.09 [-0.14, 0.32]	-0.15 [-0.45, 0.14]	0.04 [-0.16, 0.24]	0.01 [-0.13, 0.16]	0.01 [-0.16, 0.20]	0.07 [-0.11, 0.26]	-0.05 [-0.20, 0.09]	0.15 [-0.11, 0.42]
Conscientiousness	**0.43 [0.23, 0.64]*****	0.15 [-0.00, 0.31]	-0.10 [-0.26, 0.05]	-0.04 [-0.32, 0.23]	-0.01 [-0.37, 0.33]	-0.15 [-0.38, 0.08]	-0.11 [-0.32, 0.08]	-0.04 [-0.24, 0.14]	0.05 [-0.17, 0.27]	-0.11 [-0.32, 0.09]	-0.10 [-0.35, 0.15]	-0.13 [-0.37, 0.10]	-0.09 [-0.40, 0.20]	-0.02 [-0.23, 0.18]	0.03 [-0.11, 0.18]	0.03 [-0.15, 0.22]	0.00 [-0.18, 0.19]	0.06 [-0.09, 0.22]	-0.08 [-0.36, 0.18]
Openness	0.08 [-0.12, 0.30]	**0.50 [0.34, 0.67]*****	0.19 [0.02, 0.36]*	0.12 [-0.16, 0.41]	0.54 [0.18, 0.91]**	**0.40 [0.15, 0.64]*****	**0.39 [0.18, 0.61]*****	0.24 [0.03, 0.44]*	**0.49 [0.26, 0.73]*****	**0.39 [0.17, 0.61]*****	**0.61 [0.35, 0.88]*****	**0.51 [0.26, 0.76]*****	0.05 [-0.26, 0.37]	0.28 [0.06, 0.49]*	**0.34 [0.18, 0.49]*****	**0.40 [0.20, 0.60]*****	0.30 [0.10, 0.50]**	**0.31 [0.14, 0.47]*****	0.16 [-0.11, 0.45]

Notes: *: *p* <.05, **: *p* < .01, ***: *p* <= .001; Bolded font: *p* <=.001; strongest absolute association (B value) per variable is underlined.

Interpretation of English-speaking sample

In the English-speaking sample, Openness was also prominently implicated across all analyses, in consistency with the results of the Finnish sample. After controlling for the effects of other personality dimensions, Openness was found to have reliable associations with 14 DVs (all Bs > 0.24, $p \leq .001$). In addition, Openness had the strongest association with 11 DVs) – including, as in the Finnish sample, both socio-moral orientations (e.g. Other-awareness, Moral awareness, Moral Obligation) and environmental measures (e.g. NEP, Awareness of Consequences (General, Biospheric, Social & Egoistic), ECCB).

Emotionality was associated with 6 DVs (all Bs > 0.37, $p \leq .001$) including Self- and Other-awareness, IOS as well as Concern (General, Biospheric & Social). These results are very similar to those of the Finnish sample.

Extraversion was found to have a reliable association with 6 DVs (all Bs > 0.23, $p \leq .001$), including Other-awareness, IOS, and Concern (General, Social & Egoistic). It was also found to have the strongest association with IOS compared to the other dimensions as would be expected.

Honesty-Humility was found to have a reliable negative association with both Self-awareness and Egoistic Concern (all |B|s > 0.22, $p \leq .001$); this result is consistent with that of the Finnish sample.

Agreeableness was found to have a reliable association with Other-awareness and Social Concern (all Bs > 0.35, $p \leq .001$). Given that agreeable people want to please others, their care for other people's well-being seems appropriate; nonetheless, Agreeableness does not seem to be relevant for predicting other environmental attitudes. This result is somewhat different from that of the Finnish sample where no associations were found.

Conscientiousness was found to have one reliable association (B = 0.55, $p \leq .001$) with MAAS as before.

Table S.7. Multiple regression associations between HEXACO dimensions and dependent variables (English-speaking sample, N = 398)

	Mindful Attention Awareness	Social Awareness of Self	Social Awareness of Other	Inclusion of Other in Self	Inclusion of Nature in Self	Moral Awareness	Moral Obligation	Environmental Self-identity	Environmental Viewpoints
	B [95% CI]	B [95% CI]	B [95% CI]	B [95% CI]	B [95% CI]	B [95% CI]	B [95% CI]	B [95% CI]	B [95% CI]
Predictors									
Honesty-Humility	0.20 [0.03, 0.37]*	**-0.22 [-0.35, -0.09]***	0.04 [-0.09, 0.17]	-0.01 [-0.26, 0.22]	0.23 [-0.05, 0.52]	0.23 [0.01, 0.44]*	0.22 [0.04, 0.39]*	0.11 [-0.03, 0.26]	0.02 [-0.17, 0.22]
Emotionality	-0.23 [-0.40, -0.07]**	**0.37 [0.24, 0.50]***	**0.47 [0.34, 0.60]***	**0.61 [0.37, 0.85]***	-0.02 [-0.31, 0.25]	0.24 [0.03, 0.45]*	0.03 [-0.13, 0.20]	0.07 [-0.07, 0.22]	0.16 [-0.03, 0.35]
Extraversion	0.05 [-0.09, 0.19]	0.08 [-0.03, 0.19]	**0.29 [0.17, 0.40]***	**1.05 [0.84, 1.26]***	0.29 [0.04, 0.54]*	0.10 [-0.08, 0.28]	0.13 [-0.01, 0.28]	**0.23 [0.11, 0.36]***	-0.15 [-0.32, 0.01]
Agreeableness	0.15 [-0.02, 0.32]	0.11 [-0.02, 0.25]	**0.35 [0.21, 0.49]***	0.19 [-0.06, 0.45]	0.20 [-0.10, 0.51]	0.01 [-0.21, 0.24]	0.08 [-0.09, 0.27]	0.12 [-0.03, 0.29]	-0.00 [-0.21, 0.21]
Conscientiousness	**0.55 [0.39, 0.72]***	0.13 [0.00, 0.26]*	-0.15 [-0.28, -0.01]*	-0.04 [-0.28, 0.20]	0.11 [-0.17, 0.40]	0.04 [-0.17, 0.25]	0.07 [-0.09, 0.24]	0.01 [-0.12, 0.16]	0.16 [-0.03, 0.35]
Openness	0.17 [0.02, 0.32]*	**0.32 [0.20, 0.44]***	**0.24 [0.12, 0.36]***	0.10 [-0.11, 0.32]	**0.43 [0.18, 0.68]***	**0.47 [0.28, 0.66]***	**0.47 [0.32, 0.63]***	**0.38 [0.24, 0.51]***	**0.57 [0.39, 0.74]***

	General Concern	Biospheric Concern	Social Concern	Egoistic Concern	New Ecological Paradigm	Awareness of Consequences - General	Awareness of Consequences - Biospheric	Awareness of Consequences - Social	Awareness of Consequences - Egoistic	Eco-conscious Consumer Behaviour
	B [95% CI]	B [95% CI]	B [95% CI]	B [95% CI]	B [95% CI]	B [95% CI]	B [95% CI]	B [95% CI]	B [95% CI]	B [95% CI]
Predictors										
Honesty-Humility	-0.06 [-0.24, 0.10]	0.12 [-0.10, 0.34]	0.13 [-0.07, 0.34]	**-0.45 [-0.69, -0.22]***	0.26 [0.08, 0.45]**	0.12 [-0.03, 0.27]	0.16 [-0.01, 0.33]	0.10 [-0.07, 0.28]	0.09 [-0.06, 0.25]	0.14 [-0.08, 0.37]
Emotionality	**0.50 [0.33, 0.66]***	**0.51 [0.29, 0.72]***	**0.71 [0.50, 0.91]***	0.27 [0.05, 0.50]*	0.22 [0.04, 0.40]*	0.15 [0.00, 0.30]*	0.09 [-0.07, 0.26]	0.20 [0.03, 0.38]*	0.15 [0.00, 0.31]*	0.12 [-0.10, 0.34]
Extraversion	**0.29 [0.15, 0.44]***	0.05 [-0.12, 0.24]	**0.49 [0.31, 0.67]***	**0.33 [0.13, 0.53]***	-0.11 [-0.26, 0.04]	-0.12 [-0.25, 0.01]	-0.19 [-0.34, -0.05]**	-0.10 [-0.26, 0.04]	-0.05 [-0.19, 0.07]	0.20 [0.01, 0.40]*
Agreeableness	0.25 [0.06, 0.43]**	0.24 [0.01, 0.48]*	**0.40 [0.18, 0.63]***	0.09 [-0.15, 0.34]	-0.02 [-0.21, 0.17]	-0.00 [-0.17, 0.15]	-0.04 [-0.22, 0.14]	0.02 [-0.16, 0.21]	0.00 [-0.17, 0.16]	0.27 [0.03, 0.52]*
Conscientiousness	-0.00 [-0.17, 0.16]	0.07 [-0.14, 0.28]	-0.02 [-0.23, 0.18]	-0.05 [-0.28, 0.17]	0.09 [-0.08, 0.28]	0.22 [0.07, 0.38]**	0.18 [0.01, 0.35]*	0.24 [0.06, 0.42]**	0.25 [0.09, 0.41]**	0.07 [-0.15, 0.29]
Openness	0.13 [-0.02, 0.28]	**0.41 [0.22, 0.61]***	0.12 [-0.06, 0.30]	-0.13 [-0.34, 0.06]	**0.43 [0.27, 0.59]***	**0.55 [0.41, 0.68]***	**0.57 [0.42, 0.73]***	**0.51 [0.35, 0.67]***	**0.56 [0.42, 0.70]***	**0.57 [0.36, 0.77]***

Notes: *: p <.05, **: p < .01, ***: p <= .001; Bolded font: p <=.001; strongest absolute association (B value) per variable is underlined.

Summary of Supplemental Analysis

The general pattern of the results was comparable between the two samples. In both samples, Openness was the most prominent predictor of socio-moral orientations as well as environmental attitudes, although it was associated with more measures in the English-speaking population. In the Finnish sample, Openness was not associated with awareness of other people and awareness of consequences for the people, as opposed to the English-speaking sample. Furthermore, we did not find an association between Openness and eco-conscious consumerism in the Finnish sample. This is probably due to the nature of the Finnish population, which largely favours products with environmental certificates (by 40% in 2019; Hyry, 2019; close to the time-point when the data was collected), and the fact that the Finnish marketplace is characterised by high availability of eco-products (European Commission, 2019) that are easily accessible in mainstream outlets.

Both Extraversion and Emotionality showed associations with socio-moral factors that are directly related to their main characteristics such as caring for people, and feeling connected to others, as well as associations with environmental measures related to social and egoistic orientations.

Honesty-Humility had a negative association with concern about one's self in both samples, implying that those high in Honesty-Humility are less focused on themselves regarding environmental concern.

Agreeableness was associated with awareness of other people and concern for people due to environmental degradation in the English-speaking sample, while no associations were found for the Finnish sample. This can be potentially attributed to cultural differences between the samples. In both samples, Conscientiousness was only found to have one reliable association with MAAS. These results are in accordance with previous studies reporting no or weak associations of Agreeableness (Pavalache-Ilie & Cazan, 2018) and Conscientiousness (Markowitz et al., 2012; Hilbig et al., 2013) with environmental measures.

Taken together, the strong similarities between the samples indicate that the personality dimensions connect the socio-moral factors and the environmental measures in a relatively consistent manner in both populations. Although there are small differences between the samples, the general pattern of the results does not seem to change significantly due to the cultural differences. Consequently, the findings suggest that the

personality dimensions are stably associated with socio-moral orientations and environmental measures regardless of the cultural context, and underline the prominence of Openness to Experience in two distinct populations.

References

European Commission. (2019). The Environmental Implementation Review 2019. Country Report – Finland.

Hyry, J. (2019). *Kansalaiskysely ilmastonmuutoksen herättämistä tunteista ja niiden vaikutuksista kestäviin elämäntapoihin.* Kantar TNS Oy. Sitra.

Scatterplot matrix of the HEXACO dimensions

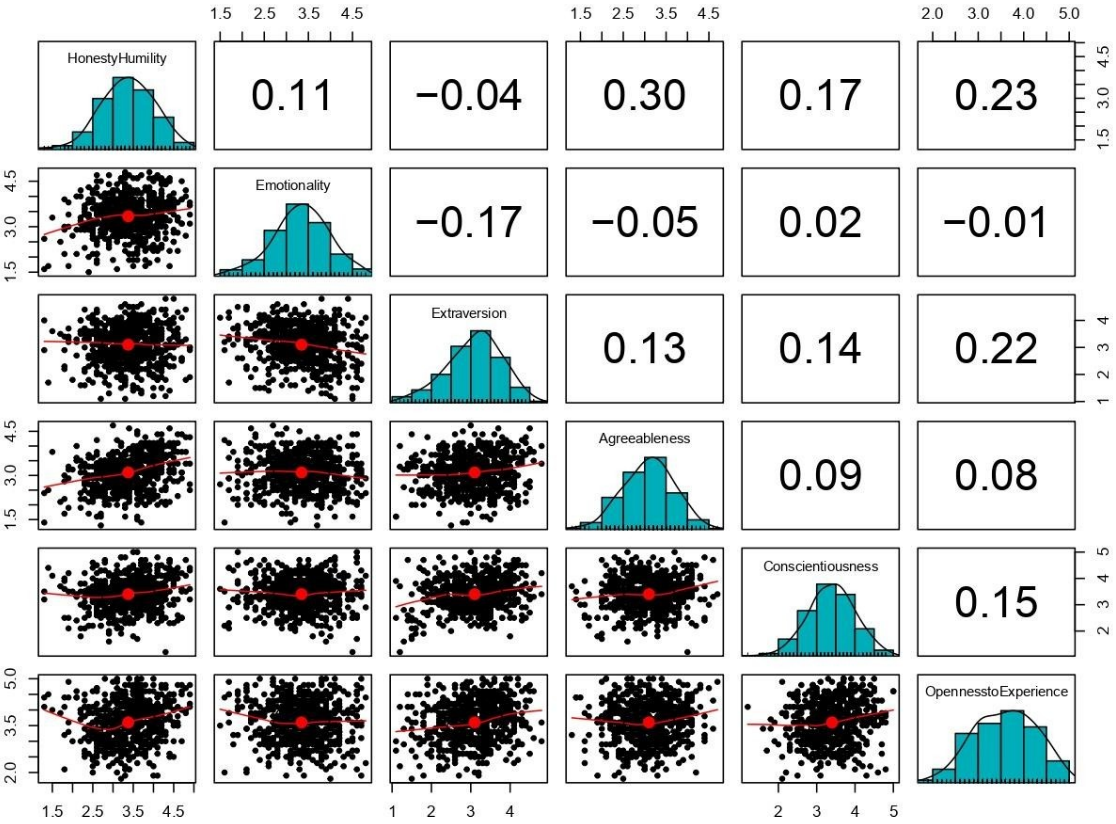

Figure S.1 depicts the histograms, correlations and scatterplots for all HEXACO dimensions.

Histograms of all dependent variables

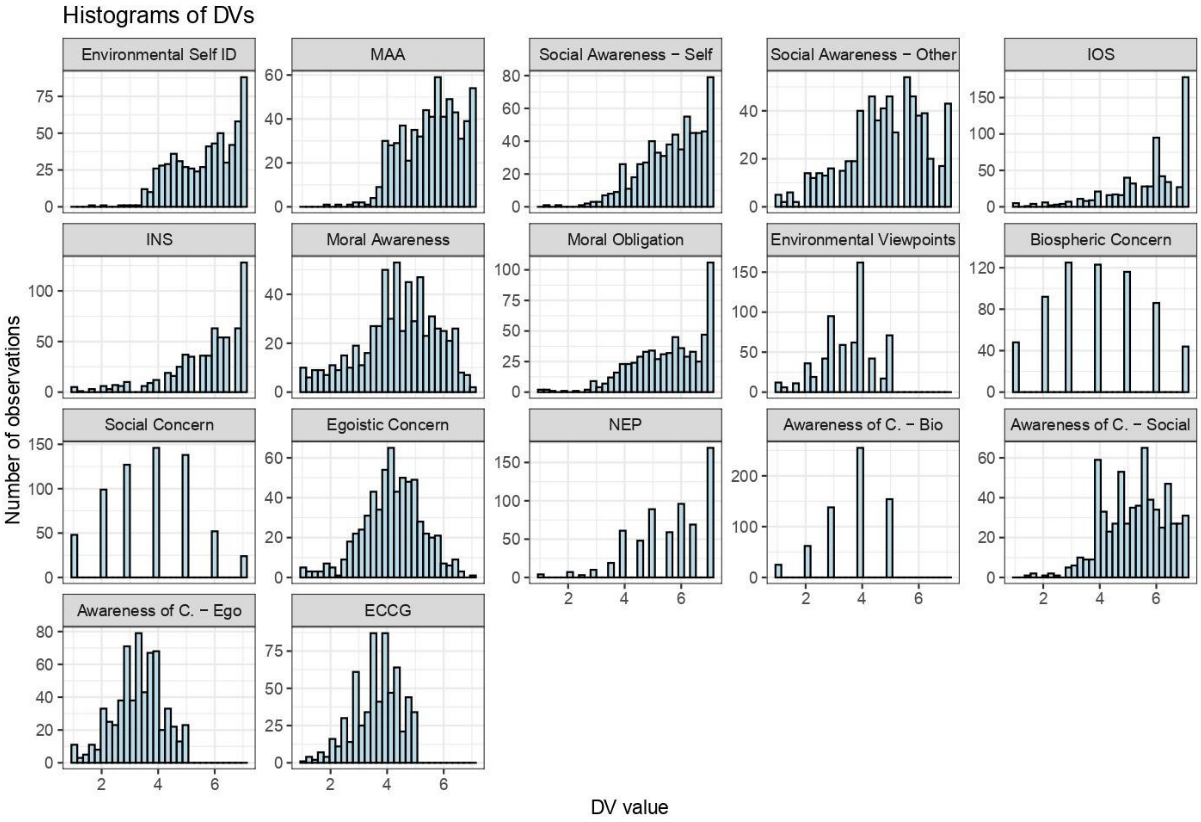

Figure S.2. depicts the histograms of all the dependent variables (excluding General concern” and “Awareness of Consequences - General) reported in the main text.

Residuals of multiple regression analyses

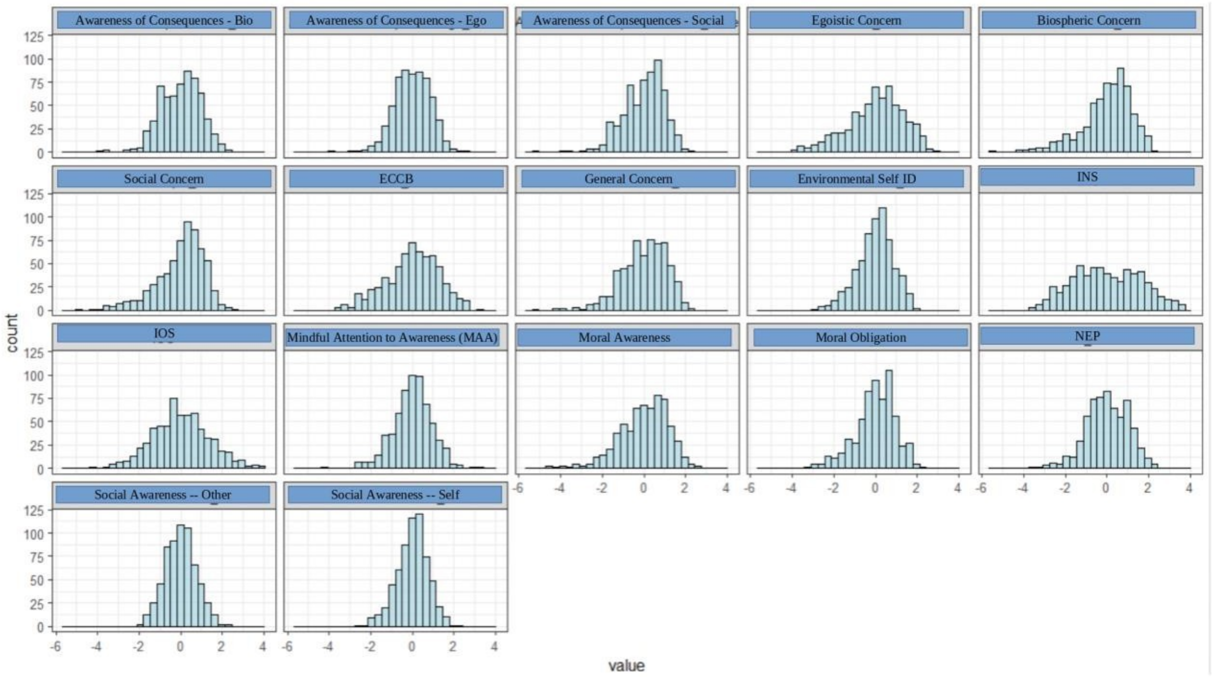

Figure S.3 depicts the residuals of all the multiple regression analyses reported in Table 2. All residuals are normally distributed.
